# Pregnancy-Induced Alterations in NK Cell Phenotype and Function

**DOI:** 10.3389/fimmu.2019.02469

**Published:** 2019-10-23

**Authors:** Mathieu Le Gars, Christof Seiler, Alexander W. Kay, Nicholas L. Bayless, Elina Starosvetsky, Lindsay Moore, Shai S. Shen-Orr, Natali Aziz, Purvesh Khatri, Cornelia L. Dekker, Gary E. Swan, Mark M. Davis, Susan Holmes, Catherine A. Blish

**Affiliations:** ^1^Department of Medicine, Stanford University, Palo Alto, CA, United States; ^2^Department of Stanford Immunology Program, Stanford University, Palo Alto, CA, United States; ^3^Department of Statistics, Stanford University, Palo Alto, CA, United States; ^4^Department of Pediatrics, Stanford University, Palo Alto, CA, United States; ^5^Faculty of Medicine, Technion - Israel Institute of Technology, Haifa, Israel; ^6^Department of Obstetrics and Gynecology, Stanford Prevention Research Center, Stanford University School of Medicine, Stanford University, Palo Alto, CA, United States; ^7^Department of Microbiology and Immunology, Stanford University, Palo Alto, CA, United States; ^8^Howard Hughes Medical Institute, Stanford University School of Medicine, Stanford, CA, United States; ^9^Chan Zuckerberg Biohub, San Francisco, CA, United States

**Keywords:** NK cells, pregnancy, influenza virus, cancer cells, NK repertoire

## Abstract

Pregnant women are particularly susceptible to complications of influenza A virus infection, which may result from pregnancy-induced changes in the function of immune cells, including natural killer (NK) cells. To better understand NK cell function during pregnancy, we assessed the ability of the two main subsets of NK cells, CD56^dim^, and CD56^bright^ NK cells, to respond to influenza-virus infected cells and tumor cells. During pregnancy, CD56^dim^ and CD56^bright^ NK cells displayed enhanced functional responses to both infected and tumor cells, with increased expression of degranulation markers and elevated frequency of NK cells producing IFN-γ. To better understand the mechanisms driving this enhanced function, we profiled CD56^dim^ and CD56^bright^ NK cells from pregnant and non-pregnant women using mass cytometry. NK cells from pregnant women displayed significantly increased expression of several functional and activation markers such as CD38 on both subsets and NKp46 on CD56^dim^ NK cells. NK cells also displayed diminished expression of the chemokine receptor CXCR3 during pregnancy. Overall, these data demonstrate that functional and phenotypic shifts occur in NK cells during pregnancy that can influence the magnitude of the immune response to both infections and tumors.

## Introduction

During pregnancy, the immune system has to finely balance its activity in order to tolerate the semi-allogeneic fetus, while maintaining the ability to fight microbial challenges (1–4). These immune alterations may be at least partially responsible for the increased susceptibility of pregnant women to complications from influenza virus infection (5–9). Recent studies have demonstrated enhanced responses to influenza virus by several innate immune cell subsets during pregnancy, including monocytes, plasmacytoid dendritic cells and natural killer (NK) cells (2, 10–14). It remains unclear whether such changes could contribute to the enhanced pathogenesis of influenza virus during pregnancy because the role of NK cells in the pathogenesis of influenza virus remains controversial. Several mouse studies have shown that NK cell depletion or the use of mice deficient in NK cells improved the outcome of influenza infection (15, 16), suggesting that NK cell activity may be pathogenic in the setting of influenza infection. On the contrary, NK cells reduced influenza virus burden and promoted clearance of the virus in mice deficient in NKp46, a major NK cell receptor thought to play a role in influenza recognition (17), suggesting that NK cells may contribute to protection from influenza. Controversy remains as another mouse strain deficient in NKp46 expression is resistant to viral infection (18). In humans, NK cells were found in abundance in the lungs of fatally infected patients with the 2009 H1N1 pandemic strain of influenza virus (19). This NK cell recruitment correlated with severity of lung inflammation and poor patient outcome, but the causality in the relation between infiltration of NK cells and viral clearance and pathogenesis is unproven.

NK cells mediate their response to influenza and other pathogens using an array of germline receptors. Inhibitory receptors serve to protect healthy cells from NK cells and include the killer-cell immunoglobulin-like receptors (KIRs) and the heterodimer NKG2A-CD94. NK cell activating receptors signal ‘altered self' and include NKp46, NKp30, NKp44, NKG2C, and NKG2D, among others. Together, the activating and inhibitory receptors define the degree of NK cell maturation and responsiveness to stimuli (20, 21). In response to virus-infected or cancerous cells, NK cells can kill cells via release of cytolytic molecules or through engagement of death receptors. They can also produce cytokines, such as IFN-γ, which limit viral replication and tumor proliferation (21). CD56^dim^ and CD56^bright^ NK cells are two major NK cell subsets identified in the peripheral blood that tend to differ in their responsiveness. CD56^dim^ NK cells are more cytotoxic and CD56^bright^ are better at secreting cytokines (22, 23). Due to their robust cytotoxic capabilities and immune regulatory potential, NK cell activation is tightly regulated to limit tissue damage at the site of infection. Here, we sought to better understand how NK cell activity is regulated during pregnancy and gain insight into the unusual susceptibility of pregnant women to complications from influenza virus and other infections. We used mass cytometry and *ex vivo* influenza infection to profile the expression of NK cell activating and inhibitory receptors during this critical period of development.

## Materials and Methods

### Study Design

Pregnant women in their second and third trimester and control non-pregnant women were enrolled in two cohorts in separate years. In the discovery cohort, twenty-one healthy pregnant women were recruited between October 2013 and March 2014 from the Obstetrics Clinic at Lucile Packard Children's Hospital at Stanford University. Twenty-one non-pregnant (control) women were recruited for Stanford influenza vaccine studies (NCT numbers: NCT03020537, NCT03022422, and NCT02141581). In the validation cohort, 32 non-pregnant (control) women were recruited for Stanford vaccine studies (NCT numbers: NCT01827462 and NCT03022422) and 21 healthy pregnant women were recruited between October 2012 and March 2013 from the Obstetrics Clinic at Lucile Packard Children's Hospital at Stanford. Venous blood was collected from all participants at baseline; pregnant women also provided a sample at 6 weeks post-partum. Exclusion criteria included concomitant illnesses, immunosuppressive medications, or receipt of blood products within the previous year. Pregnant women were also excluded for known fetal abnormalities and morbid obesity (pre-pregnancy body mass index >40). This study was performed in accordance with the Declaration of Helsinki and approved by the Stanford University Institutional Review Board (IRB-25182); written informed consent was obtained from all participants. Blood from anonymous healthy donors at the Stanford blood bank center was obtained for confirmatory functional assays.

### PBMC Isolation, Cryopreservation, and Cell Purification for Functional Assays

PBMCs from healthy donors were isolated from whole blood by Ficoll-Paque (GE Healthcare) and cryopreserved in 90% fetal bovine serum (Thermo Scientific)/10% dimethyl sulfoxide (Sigma-Aldrich). Cryopreserved PBMCs were thawed and washed with complete RP10 media [RPMI 1640 (Invitrogen) supplemented with 10% fetal bovine serum (FBS), 2 mM L-glutamine, 100 U/ml penicillin, 100 mg/ml streptomycin (Life Technologies)] and 50 U/mL benzonase (EMD Millipore). NK cells and/or monocytes were sorted using Sony sorter SH800 (Sony) with the following antibodies: CD3-Allophycocyanine (clone OKT3; BioLegend), CD14-Brilliant Violet 421 (clone HCD14; BioLegend), CD19-Alexa Fluor 488 (clone HIB19; Biolegend), and CD56-Phycoerythrin Cyanine 7 (clone NCAM; BioLegend).

### NK Cell: Infected Monocyte Co-culture

A/California/7/2009 influenza (pH1N1) wild-type influenza A virus obtained from Kanta Subbarao at the National Institutes of Health was propagated in embryonated chicken eggs. Monocytes were washed and re-suspended in serum-free RPMI media at 1 × 10^5^ per 100 μL and infected at a multiplicity of infection (MOI) of 3 for 1 h at 37°C with 5% carbon dioxide. One-hour post-infection, viral inoculum was removed and cells were resuspended in 100 μL of complete RP10. Autologous NK cells were then exposed to pH1N1-infected monocytes at a effector:target (E:T) ratio 1:1. After a further 2-h incubation, 2 μM monensin, 3 μg/mL brefeldin A (eBiosciences), and anti-CD107a-allophycocyanin-H7 (BD Pharmingen) were added to the co-culture for 4 h, followed by cell staining for flow cytometry analysis.

### K562 Cell Assay

Following purification, NK cells were exposed to K562 tumor cells (ATCC) at an effector:target (E:T) ratio of 1:1. Immediately following co-incubation, 2 μM monensin, 3 μg/mL brefeldin A, and anti-CD107a-allophycocyanin-H7 were added to the co-culture for 4 h, followed by cell staining for flow cytometry analysis.

### Cell Staining and Flow-Cytometry Analysis

Cells were stained with LIVE/DEAD fixable Aqua Stain (Life Technologies), followed by surface staining and then fixed and permeabilized with FACS Lyse and FACS Perm II (BD Pharmingen) according to the manufacturer's instructions. Cells were stained with anti-CD3-PE or -APC, anti-CD16-PerCPCy5.5 (clone 3G8; BioLegend), anti-IFNγ-FITC or V450 (clone B27; BD Biosciences), anti-CD56-PEcy7, or anti-CD14-APC or -APC-H7 and fixed using 1% paraformaldehyde. Uncompensated data were collected using a three-laser MACSQuant® Analyser (Miltenyi). Analysis and compensation were performed using FlowJo flow-cytometric analysis software, version 9.9.4 (Tree Star).

### Antibody Labeling for CyTOF

Purified antibodies (lacking carrier proteins) were labeled 100 μg at a time according to instructions provided by DVS Sciences with heavy metal-preloaded maleimide-coupled MAXPAR chelating polymers and as previously described (24, 25). Qdot antibodies purchased from Invitrogen were used for Cd112 and were not conjugated. In115, Gd155, and Gd157 were ordered from Trace Sciences and conjugated with exactly as with metals purchased from DVS Sciences. Following labeling, antibodies were diluted in PBS to a concentration between 0.1 and 0.3 mg/mL. Each antibody clone and lot was titrated to optimal staining concentrations using cell lines and primary human samples. The gating shown in Figures S2, S4, S6, S8 displays one individual as an example. Gates were set based on both positive and negative controls known to express markers, and all stains were validated by comparison to conventional flow cytometry, as described in our prior studies (20, 26). Cell subsets known to not express markers were used as negative controls in many cases (for instance, B cells do not express many NK cell markers). For some stains such as NKG2C, as new antibody conjugations and panels were used for the second cohort, the gating strategy modified if better ability to distinguish populations was possible. Gating was not used as part of the GLM analysis.

### PBMC Staining for CyTOF Acquisition

Cryopreserved PBMCs from non-pregnant and pregnant women in discovery and validation cohort were thawed and cells were transferred to 96-well deep-well-plates, resuspended in 25 μM cisplatin (Enzo Life Sciences) for 1 min and quenched with 100% serum. Cells were stained for 30 min, fixed (BD FACS Lyse), permeabilized (BD FACS Perm II), and stained with intracellular antibodies for 45 min on ice. Staining panels are described in Tables S3, S4. All antibodies were conjugated using MaxPar X8 labeling kits (DVS Sciences). Cells were suspended overnight in iridium intercalator (DVS Sciences) in 2% paraformaldehyde in phosphate-buffered saline (PBS) and washed 1 × in PBS and 2 × in H_2_O immediately before acquisition on a CyTOF-1 (Fluidigm).

### Modeling of Predictors of Pregnancy in Mass Cytometry Data

To identify markers that were consistently changed during pregnancy, we used a generalized linear model (GLM) with bootstrap resampling to account for the donor-specific heterogeneity. We implemented the GLM approach and other regression models in an open source R package *CytoGLMM* (27) available here: https://christofseiler.github.io/CytoGLMM/.

### Statistical Analysis

Linear discriminant analyses were implemented in R using the package MASS (28, 29). Statistical analyses for functional experiments were performed using GraphPad Prism, version 6.0d (GraphPad Software). A Mann-Whitney U-test was used to compare control to pregnant women and a Wilcoxon signed-rank was used to compare the paired data in women between pregnancy and the post-partum period.

### Data Availability

Mass cytometry data supporting this publication is available at ImmPort (https://www.immport.org) under study accession SDY1537.

## Results

### NK Cell Immune Response to Influenza Virus During Pregnancy

**Figure 1:**
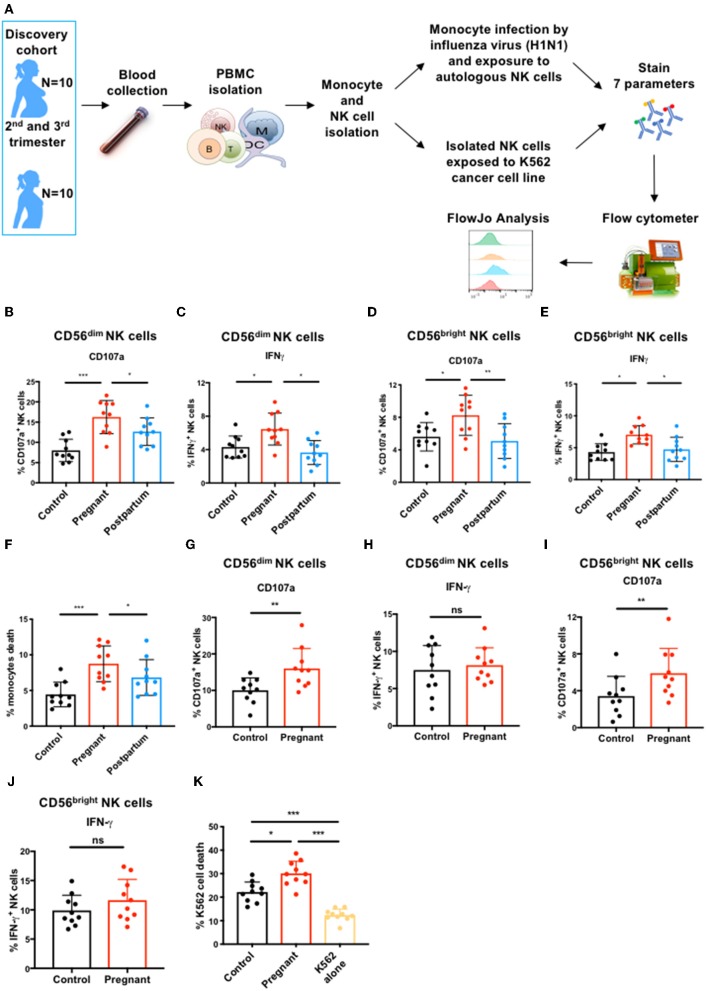
CD56^dim^ and CD56^bright^ NK cell immune response to influenza infected and tumor cells during pregnancy. **(A)** PBMCs from controls (*N* = 10), pregnant women (*N* = 10), and post-partum women (*N* = 10) in discovery cohort were isolated from blood samples. Monocytes and total NK cells were sorted and monocytes were infected with the H1N1 influenza virus strain. NK cells were either exposed to H1N1-infected monocytes or to K562 tumor cells for 7 or 4 h, respectively. **(B–I)** CD56^dim^ and CD56^bright^ NK cell immune response was then determined by flow cytometry. The frequency of **(B)** CD107a- and **(C)** IFN-γ-expressing CD56^dim^ NK cells in response to influenza-infected monocytes is represented. The frequency of **(D)** CD107a- and **(E)** IFN-γ-expressing CD56^bright^ NK cells in response to influenza-infected monocytes is represented. **(F)** The frequency of dead or dying monocytes based on staining with viability dye in NK cell co-culture. The frequency of CD107a **(G)** and IFN-γ-production **(H)** by CD56^dim^ NK cells in response to K562 cells is represented. The frequency of CD107a **(I)** and IFN-γ-production **(J)** by CD56^bright^ NK cells in response to K562 cells. **(K)** The frequency of dead or dying K562 tumor cells based on staining with viability dye in NK cell co-culture. *P < 0.05, **P < 0.01, and ***P < 0.001 (Mann–Whitney *U*-Tests to compare controls vs. pregnant; Wilcoxon matched-paired test to compare pregnant vs. post-partum).

To investigate how pregnancy alters NK cell phenotype and function, we recruited two cohorts of pregnant and non-pregnant (control) women in subsequent years (Tables S1, S2). We assessed NK cell antiviral function during pregnancy by flow cytometry after exposing sorted NK cells to autologous infected monocytes [Figure 1A; (12, 30)]. We observed that the frequency of CD56^dim^ NK cells expressing CD107a, a marker of cytolytic activity, and IFN-γ was significantly greater in pregnant women than in controls or in post-partum women (Figures 1B,C and Figure S1A). Similarly, the frequency of CD56^bright^ NK cells expressing CD107a and IFN-γ was also significantly greater during pregnancy than in controls and post-partum (Figures 1D,E and Figure S1A). Bulk NK cells from pregnant women displayed enhanced killing of influenza-infected monocytes (Figure 1F and Figure S1B). These data demonstrate that the two major NK cell subsets have enhanced responses to influenza-virus infected cells during pregnancy.

### NK Cell Immune Response to Cancer Cells During Pregnancy

During pregnancy, monocytes respond more robustly to influenza virus (11) which could activate NK cells through inflammatory cytokine production, potentially explaining enhanced NK cell responses. We hypothesized that if NK cell function was intrinsically elevated during pregnancy, we should observe enhanced anti-tumor responses as well. We therefore exposed sorted total NK cells from controls and pregnant women to the K562 tumor cell line (Figure 1A), which represents a homogenous, identical target for NK cells from controls and pregnant women. CD56^dim^ NK cells from pregnant women had 1.6-fold greater expression of CD107a than CD56^dim^ NK cells from non-pregnant women in response to K562 cells (Figure 1G and Figure S1C), though IFN-γ responses were not significantly different (Figure 1H and Figure S1C). CD56^bright^ NK cells also displayed enhanced degranulation (Figure 1I and Figure S1C) but no difference in IFN-γ production (Figure 1J and Figure S1C). This increased degranulation by both NK cell subsets from pregnant women resulted in enhanced killing of K562 cells by bulk NK cells (Figure 1K and Figure S1D). These data indicate that NK cells have an intrinsically enhanced ability to kill both infected and tumor targets during pregnancy.

### Deep Profiling of CD56^dim^ and CD56^bright^ NK Cells During Pregnancy in the Discovery Cohort

**Figure 2:**
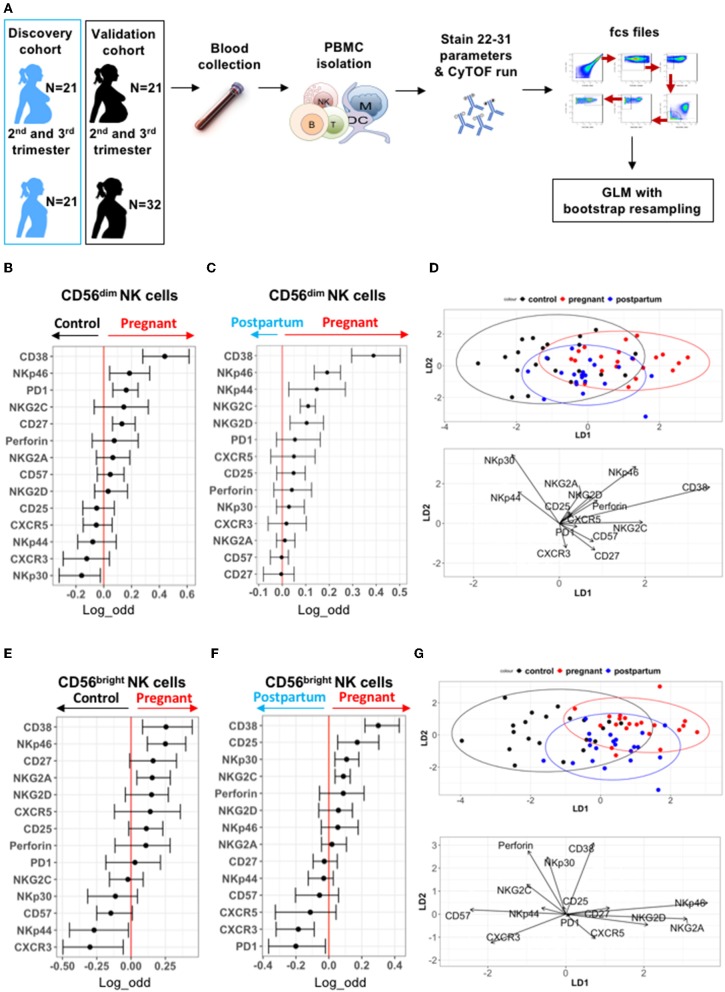
Deep profiling of CD56^dim^ and CD56^bright^ NK cells in non-pregnant and pregnant women from discovery cohort. **(A)** PBMCs from controls (*N* = 21), pregnant women (*N* = 21), and post-partum women (*N* = 21) in discovery cohort were isolated from blood samples. PBMCs were isolated and stained for mass cytometry. Data obtained were analyzed using a linear regression model, GLM. **(B,C)** Markers predictive of CD56^dim^ NK cells in control vs. pregnant women in discovery cohort were assessed by GLM with bootstrap resampling. The markers are listed on the y-axis and the x-axis represents the log-odds that the marker expression levels predict the outcome (control on the left vs. pregnancy on the right). Summary data are depicted, showing the 95% confidence interval. Markers in which the bar does not cross zero are significantly predictive of one state vs. the other. **(D)** Represents the linear discriminant analysis (LDA) for CD56^dim^ NK cells between non-pregnant, pregnant, and post-partum samples from discovery cohort. **(E,F)** Markers predictive of CD56^bright^ NK cells in control vs. pregnant women in discovery cohort were assessed by GLM with bootstrap resampling. The markers are listed on the y-axis and the x-axis represents the log-odds that the marker expression levels predict the outcome (control on the left vs. pregnancy on the right). Summary data are depicted, showing the 95% confidence interval. Markers in which the bar does not cross zero are significantly predictive of one state vs. the other. **(G)** Represents the linear discriminant analysis (LDA) for CD56^bright^ NK cells between non-pregnant, pregnant, and post-partum samples from discovery cohort.

To understand potential drivers of this enhanced NK cell function during pregnancy, we profiled the expression patterns of inhibitory and activating surface receptors on NK cells in control non-pregnant women, pregnant women, and post-partum women (including the 10 individuals per group tested in Figure 1). PBMCs in both cohorts were evaluated by mass cytometry as outlined in Figure 2A and Tables S3, S4. NK cells were identified as CD3^−^CD19^−^CD20^−^CD14^−^CD56^dim/bright^CD16^+/−^ cells (Figure S2A). The frequency of NK cells did not significantly differ between pregnant and control women, nor in pregnant vs. post-partum women in either cohort (Figures S2B,C). To identify NK cell markers predictive of pregnancy, we used a Generalized Linear Model (GLM) with bootstrap resampling to account for correlations between cells and inter-individual variability [Figure 2A; (27)].

Expression of several markers such as CD38, NKp46, PD-1, and CD27 were predictive of pregnancy on CD56^dim^ NK cells, while NKp30 was more likely to predict control (Figure 2B). When comparing the same women during pregnancy and post-partum, CD38, NKp46, NKG2C, NKG2D, and NKp44 were predictive of pregnancy on CD56^dim^ NK cells (Figure 2C). Manual gating confirmed elevated expression of CD38 and NKp46 on CD56^dim^ NK cells during pregnancy (Figures S3, S4). To further define the markers that distinguish pregnancy, a linear discriminant analysis (LDA) was performed, revealing that CD38 and NKp46 best separate the CD56^dim^ NK cell population of pregnant women from that of control and post-partum women (Figure 2D). Together, our data indicate that there are differences in NK receptor expression patterns during pregnancy, and that CD38 and NKp46 expression are major drivers of these pregnancy-related changes.

As CD56^bright^ NK cells differ from CD56^dim^ NK cells in their maturation and receptor expression patterns, we analyzed them separately. CD38 and NKp46 expression levels are also predictive of pregnancy on CD56^bright^ NK cells, as is the inhibitory receptor NKG2A, which is highly expressed on CD56^bright^ NK cells (Figure 2E and Figures S5, S6). Expression of the chemokine receptor, CXCR3, and activating receptor, NKp44, were associated with non-pregnant state. Similar differences were seen when comparing pregnant and post-partum samples (Figure 2F and Figures S5, S6). LDA reveals that CD38, NKp46, NKG2A, and NKG2D best separate CD56^bright^ NK cells of pregnant women from that of control and post-partum women (Figure 2G). Together, these data suggest that during pregnancy, both CD56^dim^ and CD56^bright^ NK cell subsets have the potential for greater activation through an increased expression of CD38 and NKp46.

### Deep Profiling of CD56^dim^ and CD56^bright^ NK Cells in the Validation Cohort

**Figure 3:**
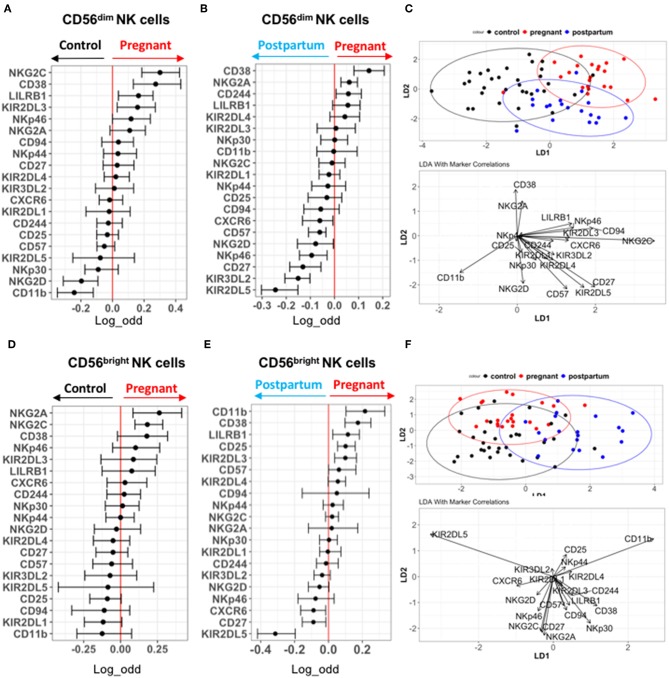
Deep profiling of CD56^dim^ and CD56^bright^ NK cells in non-pregnant and pregnant women from validation cohort. **(A,B)** Markers predictive of CD56^dim^ NK cells in control vs. pregnant women in validation cohort were assessed by GLM with bootstrap resampling. The markers are listed on the y-axis and the x-axis represents the log-odds that the marker expression levels predict the outcome (control on the left vs. pregnancy on the right). Summary data are depicted, showing the 95% confidence interval. Markers in which the bar does not cross zero are significantly predictive of one state vs. the other. **(C)** Represents the linear discriminant analysis (LDA) for CD56^dim^ NK cells between non-pregnant, pregnant and post-partum samples from validation cohort. **(D,E)** Markers predictive of CD56^bright^ NK cells in control vs. pregnant women in validation cohort were assessed by GLM with bootstrap resampling. The markers are listed on the y-axis and the x-axis represents the log-odds that the marker expression levels predict the outcome (control on the left vs. pregnancy on the right). Summary data are depicted, showing the 95% confidence interval. Markers in which the bar does not cross zero are significantly predictive of one state vs. the other. **(F)** Represents the linear discriminant analysis (LDA) for CD56^bright^ NK cells between non-pregnant, pregnant and post-partum samples from validation cohort.

We performed a deeper profiling of NK cells in the validation cohort, using an antibody panel including an increased number of specific NK cell receptors such as KIRs (Figure 2A and Table S4). Similar to the discovery cohort, CD38 and NKp46 are predictive of pregnancy on CD56^dim^ NK cells compared to controls (Figure 3A). CD56^dim^ NK cells from pregnant women also display an increased expression of NKG2C, LILRB1, and KIR2DL3 compared to control, while NKG2D and CD11b expression predicted control. CD38, NKG2A, and CD244 expression are also predictive of pregnancy when compared with post-partum conditions, while several markers including KIRs predicted the post-partum state (Figure 3B). NKp46 predicted the post-partum state among CD56^dim^ NK cells in the validation cohort (Figure 3B). Manual gating confirmed the results of the GLM for this cohort (Figures S7, S8). LDA performed on these data showed that CD38 and NKG2A best explained the separation between CD56^dim^ NK cells from pregnant women with controls and post-partum in the validation cohort (Figure 3C).

For CD56^bright^ NK cells, CD11b, CD38, LILRB1, CD25, KIR2DL3, NKG2A, and NKG2C are predictive of pregnancy, while several markers predict the post-partum state (Figures 3D,E). These data were confirmed by manual gating (Figures S9, S10). LDA separation showed that CD38, NKp30, CD94, and CD244 most contribute to the separation of CD56^bright^ NK cells from pregnant women compared to controls and post-partum (Figure 3F). Several markers differed in their predictions between the discovery and validation cohorts. For instance, NKG2C was predictive of pregnancy in comparison to control among both CD56^dim^ and CD56^bright^ NK cells in the validation cohort, but not in the discovery cohort. This raises the possibility that there are differences in CMV status between cohorts driving the effect. Unfortunately, CMV serologies were not available; however, there were no significant differences in the frequency of “adaptive” NKG2C^+^CD57^+^ NK cells between the control, pregnant, or post-partum women in either cohort, making it less likely that differences in CMV status were driving the differences in NK cell phenotype (Figure S11). Overall, the most consistent finding in pregnancy is the increased expression of CD38 on both CD56^dim^ and CD56^bright^ NK cells. There is significant variation in the expression patterns of activating and inhibitory NK cell receptors during pregnancy, but pregnancy is associated with a higher activation status and enhanced CD38 expression.

### Co-expression of CD38 and NKp46

**Figure 4:**
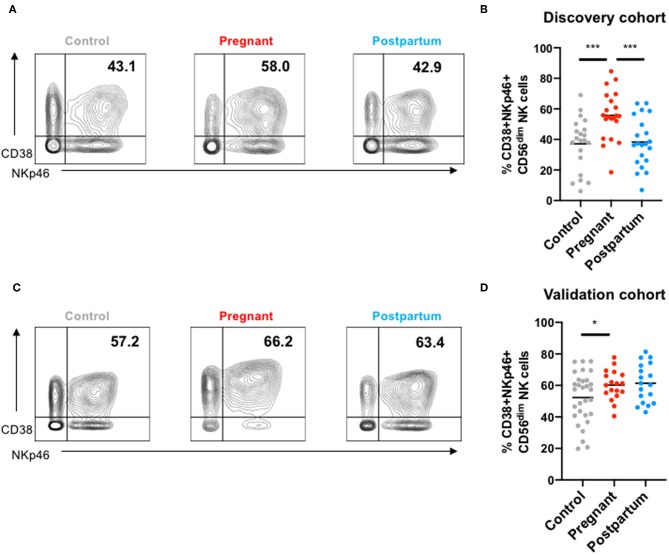
NKp46 and CD38 co-expression in NK cells from controls, pregnant and post-partum women in the discovery and validation cohort. **(A)** Representative mass cytometry plots showing the co-expression of NKp46 and CD38 in peripheral NK cells from a control, pregnant or post-partum women in the discovery cohort. **(B)** Frequency of CD38^+^NKp46^+^ NK cells from controls, pregnant and post-partum individuals in the discovery cohort. ***P < 0.001 (Mann–Whitney *U*-Tests to compare controls vs. pregnant; Wilcoxon matched-paired test to compare pregnant vs. post-partum). **(C)** Representative mass cytometry plots showing the co-expression of NKp46 and CD38 in peripheral NK cells from a control, pregnant, or post-partum women in the validation cohort. **(D)** Frequency of CD38^+^NKp46^+^ NK cells from controls, pregnant and post-partum individuals in the validation cohort. *P < 0.05 (Mann–Whitney *U*-Tests to compare controls vs. pregnant; Wilcoxon matched-paired test to compare pregnant vs. post-partum).

As the most consistently observed difference was enhanced expression of CD38 and NKp46 on CD56^dim^ NK cells during pregnancy, we examined the frequency of CD56^dim^ NK cells co-expressing these markers (Figure 4). CD38 and NKp46 were co-expressed on a greater frequency of NK cells both the discovery (Figures 4A,B) and validation cohorts during pregnancy (Figures 4C,D). In the discovery cohort, the frequency of CD38^high^NKp46^+^ NK cells returned to levels found in controls during the post-partum period, but in the validation cohort, the frequency of CD38^high^NKp46^+^ NK cells remained high in the post-partum period. There was no significant association between the frequency of CD38^high^NKp46^+^ NK cells and “adaptive” NKG2C^+^CD57^+^ NK cells (Figure S11).

## Discussion

During pregnancy, the maternal immune system is engaged in a fine balance: tolerance is required to preserve the fetus while defenses must be maintained to protect mother and baby from microbial challenges. NK cells play a critical role in this balance as their job is to patrol the body for “altered self” (31). NK cell activity had been thought to be suppressed during pregnancy to protect the fetus, but recent studies have suggested a more nuanced view (2). NK cells from pregnant women display diminished responses to stimulation with cytokines and phorbol-myristate acetate and ionomycin, yet NK cell responses to influenza-infected cells are enhanced (12–14, 32). Here we show that both CD56^dim^ and CD56^bright^ NK cell subsets have enhanced responses to both the influenza virus and to cancer cells, indicating a cell-intrinsic enhancement in their response to threats. Profiling CD56^dim^ and CD56^bright^ NK cells from pregnant and non-pregnant women showed that during pregnancy, both subsets are characterized by increased expression of the activation marker, CD38. CD38 is expressed on a large proportion of NK cells even in non-pregnant individuals and is significantly increased in cell surface density during pregnancy. CD56^dim^ NK cells also demonstrate increased expression of the activating receptor NKp46 during pregnancy (though it is even higher in the post-partum period in one study); this receptor may play a role in recognition of influenza-infected cells (33, 34). These observations indicate that NK cells have an enhanced expression of receptors that mark NK cell activation and contribute to the response to influenza virus and cancer cells.

Pregnant women are significantly more likely to suffer adverse consequences from influenza infection than are the general population. During the 1918 influenza pandemic, the case fatality rate for influenza infection was estimated to be 27–75% among pregnant women but only 2–3% among the general population (35). Even with improved supportive care, the case-fatality rate among pregnant women was twice that of the general population during the 2009 pandemic (36). Thus, an understanding of the mechanisms driving this enhanced susceptibility to influenza infection during pregnancy represents an important challenge for the scientific community. During influenza virus infection, the recruitment of peripheral NK cells into the lungs represents one of the first lines of defense following influenza infection (37). Though isolated NK cells stimulated with cytokines or chemicals have suppressed responses during pregnancy, our data here confirm earlier findings that NK cell responses to autologous influenza-infected cells are enhanced during pregnancy (12). This enhanced responsiveness could be deleterious to lung integrity and drive pathogenesis. Consistent with this idea, Kim et al. demonstrated that pregnant mice infected by influenza virus have increased lung inflammation and damage compared to non-pregnant mice (38). Further, Littauer et al. suggested that innate immune responses play a role in the initiation of pregnancy complications such as preterm birth and stillbirth following influenza virus infection (5). Finally, the idea that enhanced NK cell responses could be detrimental in pregnant women is consistent with observations that hyperinflammatory responses are a driving force behind severe influenza disease in humans (39–41).

To deepen our understanding of the effect of pregnancy on NK cell responses, we turned to mass cytometry to profile the expression of NK cell surface receptors. We were surprised to discover that both CD56^dim^ and CD56^bright^ NK cell subsets had a consistent and significant increase in CD38 expression during pregnancy compared to non-pregnant and post-partum samples. While CD38 is commonly viewed as an activation marker on T cells, it is more highly expressed on NK cells and has several important functions. First, CD38 confers lymphocytes with the ability to adhere to endothelial cells through its binding to CD31, a necessary step in extravasation. CD38 also functions as an ectoenzyme, converting extracellular NAD^+^ to cADPR through its cyclase activity or cADPR to Adenosyl-di-phosphate ribose through its hydrolase activity (42). These molecules in turn can diffuse into the cell and promote its activation by driving intracellular calcium increase, phosphorylation of signaling molecules, production of cytokines, and vesicular transport (43). CD38 crosslinking can enhance the cytotoxic activity of cytokine-activated NK cells (44–46) and plays a role in immune synapse formation in T cells (47) and NK cells (Le Gars et al., unpublished data). Thus, this increased CD38 expression during pregnancy might explain the enhanced responses of NK cells to influenza and tumor cells. Interestingly, decidual NK cells express high levels of CD38 compared to peripheral NK cells, yet their origin is still unclear (48). It has been proposed that subsets of NK cells can migrate from the maternal blood to the decidua and acquire the unique features of decidual NK cells upon exposure to decidual environment (49, 50). Our data suggest that the overall environment during pregnancy could enhance CD38 expression. Several studies suggest that KIR2DL4 could play a significant role in regulating IFN-γ production by decidual NK cells (51–53). Further, an NK cell population found in repeated pregnancies, which has a unique transcriptome and epigenetic signature, is characterized by high expression of the receptors NKG2C and LILRB1 (54). This NK cell population has open chromatin around the enhancers of IFNG and VEGF genes, which leads to an increased production of IFN-γ and VEGF upon activation. This is consistent with our finding that NKG2C and LILRB1 expression is increased in our validation cohort, and could explain the increased activation of peripheral NK cells upon encounter with infected or tumor cells during pregnancy.

Another interesting finding is the consistent increased expression of NKp46 on CD56^dim^ NK cells during pregnancy. Intriguingly, in the validation cohort, NKp46 levels were even higher on CD56^dim^ NK cells during the post-partum period. NKp46 has been shown to contribute to NK cell influenza virus responses through binding of influenza hemagglutinin (34). Signaling mediated by NKp46 following influenza sensing leads to the production of IFN-γ (33, 55). Therefore, an increased expression of NKp46 during pregnancy could make NK cells more responsive to influenza virus. Further, more elevated expression of NKp46 facilitates the control of lung cancer in mice (56) and NKp46 alteration is associated with tumor progression in human gastric cancer (57). Thus, the increased expression of NKp46 on CD56^dim^ NK cells, together with CD38, could explain the enhanced response to cancer cells during pregnancy. Two factors limited our ability to directly attribute the enhanced expression of CD38 and NKp46 to NK cell hyperresponsiveness during pregnancy. First, we did not have sufficient PBMC samples from pregnant women to perform blocking experiments. Second, even with enough material, CD38 and NKp46 are expressed on NK cells from non-pregnant women as well, albeit at lower levels, thus blocking would be expected to diminish responses in both pregnant and non-pregnant women.

Several markers differed in their expression pattern during pregnancy in only one cohort, and there was significant variation in the expression patterns of some markers between cohorts. For instance, NKG2D cells was predictive of pregnancy in the discovery cohort and predictive of post-partum/control in the validation cohort. This may reflect the substantial differences in NK cell phenotype between individuals. In earlier work we noted that NK cell receptor expression profiles, particularly those of activating receptors, differed dramatically between identical twins and based on maturation status, suggesting that these expression patterns are influenced by the environment (20, 26). This high variation between individuals may also explain our failure to observe consistent pregnancy-related changes in NKp30 or NKp44 expression. Other changes associated with pregnancy, including expression patterns of LILRB1, KIR3DL2, and KIR2DL5 were only evaluated in the validation cohort and warrant follow-up in future studies. Changes in NKG2C expression observed on CD56^dim^ NK cells between the pregnant and control subjects could reflect differences in the CMV status between the cohorts in the cross-sectional analyses; unfortunately, CMV status is not known for the cohorts. An additional feature that was observed in the discovery cohort, but unfortunately not evaluated in the validation cohort, was decreased expression of CXCR3 on CD56^bright^ NK cells during pregnancy. CXCR3, through the binding to its ligand IP-10, is an important receptor responsible for the recruitment of NK cells to the site of infection or inflammation. The CXCR3/IP-10 axis has been shown to enhance acute respiratory distress syndrome (ARDS) by the increased systemic presence of IP-10 (58). Thus, decreased level of CXCR3 on NK cells fails to explain their enhanced responses during pregnancy but could represent a mechanism of protection to avoid an excessive recruitment of CD56^bright^ NK cells to the lung of influenza-infected pregnant women and to restrain lung damage.

Why does NK cell phenotype undergo such changes during pregnancy? The answer remains unclear. The presence of fetal antigens in maternal blood could explain the increased activation state of NK cells. Monocytes and dendritic cells exert a pro-inflammatory phenotype during pregnancy (2, 11, 14) and this could be in part due to parental antigens present in the fetus. In turn, monocytes and pDCs could produce several cytokines such as IL-15,−18, or IFN-type I to promote increased NK cell receptor expression and activate NK cells (59). Another possibility to explain the observed phenotypic changes of NK cells during pregnancy is hormonal variation. These fluctuations could promote transcriptomic and epigenetic modifications driving alteration of NK cell phenotype and response to influenza virus and tumor cells. However, several studies suggest that progesterone and estrogen dampen NK cell cytotoxic activities (60, 61). A deep analysis of the transcriptomic and epigenetic landscape of NK cells during pregnancy could lead to a better understanding of these NK cell changes.

There are several limitations of our study, including the fact that our mass cytometry panels differed between the two cohorts and remain limited to ~40 markers. Thus, we may have excluded other molecules involved in NK cell immune responses during pregnancy, including critical NK cell surface molecules such as DNAM-1, TIGIT, and Siglec-7. We also did not follow-up on other differences that were seen in only one cohort. Further, here we studied peripheral blood NK cells and were not able to sample lung resident NK cells or uterine NK cells. Finally, we had limited data reflecting the history of the pregnant and control women in terms of their prior vaccination status, prior influenza infection status, cigarette and drug use, and others. We cannot exclude that unmeasured factors could influence the NK cell phenotype and the quality of the NK cell responses to influenza and cancer cells.

Here, our goal was to refine current understanding of NK cell biology and activity in the context of pregnancy and influenza virus infection. Our work reveals enhanced activity of both CD56^dim^ and CD56^bright^ NK cell subsets to influenza-infected cells and tumor cells during pregnancy. These enhanced responses are associated with a more robust expression of CD38, a receptor that plays a role in activation and cytotoxicity, and NKp46, a receptor associated with a better response to influenza virus and certain cancers. Together, our data provide a more complete view of the immune changes mediated by pregnancy and enhances our understanding of the susceptibility of pregnant women to influenza virus.

## Data Availability Statement

Mass cytometry data supporting this publication is available at ImmPort (https://www.immport.org) under study accession SDY1537.

## Ethics Statement

The studies involving human participants were reviewed and approved by Stanford University Institutional Review Board. The patients/participants provided their written informed consent to participate in this study. Written informed consent was obtained from the individual(s) for the publication of any potentially identifiable images or data included in this article.

## Author Contributions

ML, AK, NB, and CB designed experiments. ML, AK, and NB, and CS analyzed the data. LM, SS-O, and PK collaborated and provided advice in the analysis of the data. MD, CD, GS, and NA coordinated and provided human samples. ML and CB wrote the manuscript. All authors contributed revisions and edits.

### Conflict of Interest

The authors declare that the research was conducted in the absence of any commercial or financial relationships that could be construed as a potential conflict of interest.

## References

[1] Erlebacher A. Immunology of the maternal-fetal interface. Ann Rev Immunol. (2013) 31:387–411. 10.1146/annurev-immunol-032712-100003 23298207

[2] Kourtis AP Read JS Jamieson DJ. Pregnancy and infection. N Engl J Med. (2014) 370:2211–8. 10.1056/NEJMra1213566 24897084 PMC4459512

[3] Periolo N Avaro M Czech A Russo M Benedetti E Pontoriero A. Pregnant women infected with pandemic influenza A(H1N1)pdm09 virus showed differential immune response correlated with disease severity. J Clin Virol. (2015) 64:52–8. 10.1016/j.jcv.2015.01.009 25728079

[4] PrabhuDas M Bonney E Caron K Dey S Erlebacher A Fazleabas A. Immune mechanisms at the maternal-fetal interface: perspectives and challenges. Nat Immunol. (2015) 16:328–34. 10.1038/ni.3131 25789673 PMC5070970

[5] Littauer EQ Esser ES Antao OQ Vassilieva EV Compans RW Skountzou I. H1N1 influenza virus infection results in adverse pregnancy outcomes by disrupting tissue-specific hormonal regulation. PLoS Pathogens. (2017) 13:e1006757. 10.1371/journal.ppat.1006757 29176767 PMC5720832

[6] Pazos M Sperling RS Moran TM Kraus TA. The influence of pregnancy on systemic immunity. Immunol Res. (2012) 54:254–61. 10.1007/s12026-012-8303-9 22447351 PMC7091327

[7] Omer SB Bednarczyk R Madhi SA Klugman KP. Benefits to mother and child of influenza vaccination during pregnancy. Hum Vacc Immunother. (2012) 8:130–7. 10.4161/hv.8.1.18601 22251998

[8] Raj RS Bonney EA Phillippe M. Influenza, immune system, and pregnancy. Reprod Sci. (2014) 21:1434–51. 10.1177/1933719114537720 24899469 PMC4231130

[9] Siston AM Rasmussen SA Honein MA Fry AM Seib K Callaghan WM. Pandemic 2009 influenza A(H1N1) virus illness among pregnant women in the United States. JAMA. (2010) 303:1517–25. 10.1001/jama.2010.479 20407061 PMC5823273

[10] Aghaeepour N Ganio EA Mcilwain D Tsai AS Tingle M Van Gassen S. An immune clock of human pregnancy. Sci Immunol. (2017) 2:eaan2946. 10.1126/sciimmunol.aan2946 28864494 PMC5701281

[11] Gars ML Le Gars M Kay AW Bayless NL Aziz N Dekker CL. Increased proinflammatory responses of monocytes and plasmacytoid dendritic cells to influenza A virus infection during pregnancy. J Infect Dis. (2016) 214:1666–71. 10.1093/infdis/jiw448 27655870 PMC5144734

[12] Kay AW Fukuyama J Aziz N Dekker CL Mackey S Swan GE. Enhanced natural killer-cell and T-cell responses to influenza A virus during pregnancy. Proc Natl Acad Sci USA. (2014) 111:14506–11. 10.1073/pnas.1416569111 25246558 PMC4210016

[13] Kraus TA Engel SM Sperling RS Kellerman L Lo Y Wallenstein S. Characterizing the pregnancy immune phenotype: results of the viral immunity and pregnancy (VIP) study. J Clin Immunol. (2012) 32:300–11. 10.1007/s10875-011-9627-2 22198680 PMC7086597

[14] Kraus TA Sperling RS Engel SM Lo Y Kellerman L Singh T. Peripheral blood cytokine profiling during pregnancy and post-partum periods. Am J Reprod Immunol. (2010) 64:411–26. 10.1111/j.1600-0897.2010.00889.x 20712812

[15] Abdul-Careem MF Mian MF Yue G Gillgrass A Chenoweth MJ Barra NG. Critical role of natural killer cells in lung immunopathology during influenza infection in mice. J Infect Dis. (2012) 206:167–77. 10.1093/infdis/jis340 22561366

[16] Zhou G Juang SWW Kane KP. NK cells exacerbate the pathology of influenza virus infection in mice. Eur J Immunol. (2013) 43:929–38. 10.1002/eji.201242620 23436540

[17] Gazit R Gruda R Elboim M Arnon TI Katz G Achdout H. Lethal influenza infection in the absence of the natural killer cell receptor gene Ncr1. Nat Immunol. (2006) 7:517–23. 10.1038/ni1322 16565719

[18] Narni-Mancinelli E Jaeger BN Bernat C Fenis A Kung S De Gassart A. Tuning of natural killer cell reactivity by NKp46 and Helios calibrates T cell responses. Science. (2012) 335:344–8. 10.1126/science.1215621 22267813

[19] Mauad T Hajjar LA Callegari GD da Silva LFF Schout D Galas FRBG. Lung pathology in fatal novel human influenza A (H1N1) infection. Am J Respirat Critic Care Med. (2010) 181:72–9. 10.1164/rccm.200909-1420OC 19875682

[20] Strauss-Albee DM Horowitz A Parham P Blish CA. Coordinated regulation of NK receptor expression in the maturing human immune system. J Immunol. (2014) 193:4871–9. 10.4049/jimmunol.1401821 25288567 PMC4225175

[21] Vivier E Tomasello E Baratin M Walzer T Ugolini S. Functions of natural killer cells. Nat Immunol. (2008) 9:503–10. 10.1038/ni1582 18425107

[22] Cooper MA Fehniger TA Turner SC Chen KS Ghaheri BA Ghayur T Human natural killer cells: a unique innate immunoregulatory role for the CD56bright subset. Blood. (2001) 97:3146–51. 10.1182/blood.V97.10.3146 11342442

[23] Nagler A Lanier LL Cwirla S Phillips JH. Comparative studies of human FcRIII-positive and negative natural killer cells. J Immunol. (1989) 143:3183–91. 2530273

[24] Kay AW Strauss-Albee DM Blish CA. Application of mass cytometry (CyTOF) for functional and phenotypic analysis of natural killer cells. Methods Mol Biol. (2016) 1441:13–26. 10.1007/978-1-4939-3684-7_2 27177653 PMC5304457

[25] Strauss-Albee DM Blish CA CyTOF: single cell mass cytometry for evaluation of complex innate cellular phenotypes. In: Montgomery R Bucala R, editors. Experimental Approaches for the Investigation of Innate Immunity. World Scientific; Yale University School of Medicine (2015). p. 27–39. 10.1142/9789814678735_0003

[26] Horowitz A Strauss-Albee DM Leipold M Kubo J Nemat-Gorgani N Dogan OC. Genetic and environmental determinants of human NK cell diversity revealed by mass cytometry. Sci Transl Med. (2013) 5:208ra145. 10.1126/scitranslmed.3006702 24154599 PMC3918221

[27] Seiler C Kronstad LM Simpson LJ Le Gars M Vendrame E Blish CA Uncertainty Quantification in Multivariate Mixed Models for Mass Cytometry Data. (2019). Retrieved from: http://arxiv.org/abs/1903.07976 (accessed October 15, 2019).

[28] Gentleman R Carey V Huber W Irizarry R Dudoit S Bioinformatics and Computational Biology Solutions Using R and Bioconductor. Springer Science & Business Media (2006). 10.1007/0-387-29362-0

[29] Ripley B Venables B Bates DM Hornik K Gebhardt A Firth D MASS: Support Functions and Datasets for Venables and Ripley's MASS. R Package Version 7–3. (2011).

[30] Kronstad LM Seiler C Vergara R Holmes SP Blish CA. Differential induction of IFN-α and modulation of CD112 and CD54 expression govern the magnitude of NK cell IFN-γ response to influenza A viruses. J Immunol. (2018) 201:2117–31. 10.4049/jimmunol.1800161 30143589 PMC6143432

[31] Hilton HG Parham P. Missing or altered self: human NK cell receptors that recognize HLA-C. Immunogenetics. (2017) 69:567–79. 10.1007/s00251-017-1001-y 28695291 PMC5560170

[32] Veenstra van Nieuwenhoven AL Bouman A Moes H Heineman MJ de Leij LFMH Santema J Cytokine production in natural killer cells and lymphocytes in pregnant women compared with women in the follicular phase of the ovarian cycle. Fertil Steril. (2002) 77:1032–7. 10.1016/S0015-0282(02)02976-X 12009363

[33] Glasner A Zurunic A Meningher T Lenac Rovis T Tsukerman P Bar-On Y. Elucidating the mechanisms of influenza virus recognition by Ncr1. PLoS ONE. (2012) 7:e36837. 10.1371/journal.pone.0036837 22615821 PMC3352933

[34] Mandelboim O Lieberman N Lev M Paul L Arnon TI Bushkin Y. Recognition of haemagglutinins on virus-infected cells by NKp46 activates lysis by human NK cells. Nature. (2001) 409:1055–60. 10.1038/35059110 11234016

[35] Rasmussen SA Jamieson DJ Bresee JS. Pandemic influenza and pregnant women. Emerg Infect Dis. (2008) 14:95–100. 10.3201/eid1401.070667 18258087 PMC2600164

[36] Centers for Disease Control and Prevention (CDC) Estimates of deaths associated with seasonal influenza — United States, 1976-2007. MMWR. MMWR Morb Mortal Wkly Rep. (2010) 59:1057–62. 20798667

[37] Carlin LE Hemann EA Zacharias ZR Heusel JW Legge KL. Natural killer cell recruitment to the lung during influenza A virus infection is dependent on CXCR3, CCR5, and virus exposure dose. Front Immunol. (2018) 9:781. 10.3389/fimmu.2018.00781 29719539 PMC5913326

[38] Kim HM Kang YM Song BM Kim HS Seo SH. The 2009 pandemic H1N1 influenza virus is more pathogenic in pregnant mice than seasonal H1N1 influenza virus. Viral Immunol. (2012) 25:402–10. 10.1089/vim.2012.0007 22985287

[39] Cheung CY Poon LLM Lau AS Luk W Lau YL Shortridge KF. Induction of proinflammatory cytokines in human macrophages by influenza A (H5N1) viruses: a mechanism for the unusual severity of human disease? Lancet. (2002) 360:1831–7. 10.1016/S0140-6736(02)11772-7 12480361

[40] de Jong MD Simmons CP Thanh TT Hien VM Smith GJD Chau TNB. Fatal outcome of human influenza A (H5N1) is associated with high viral load and hypercytokinemia. Nat Med. (2006) 12:1203–7. 10.1038/nm1477 16964257 PMC4333202

[41] Kobasa D Jones SM Shinya K Kash JC Copps J Ebihara H. Aberrant innate immune response in lethal infection of macaques with the 1918 influenza virus. Nature. (2007) 445:319–23. 10.1038/nature05495 17230189

[42] Deaglio S Mallone R Baj G Arnulfo A Surico N Dianzani U. CD38/CD31, a receptor/ligand system ruling adhesion and signaling in human leukocytes. Chem Immunol. (2000) 75:99–120. 10.1159/000058765 10851781

[43] Lee HC. Structure and enzymatic functions of human CD38. Mol Med. (2006) 12:317–23. 10.2119/2006-00086.Lee 17380198 PMC1829193

[44] Deaglio S Zubiaur M Gregorini A Bottarel F Ausiello CM Dianzani U. Human CD38 and CD16 are functionally dependent and physically associated in natural killer cells. Blood. (2002) 99:2490–8. 10.1182/blood.V99.7.2490 11895784

[45] Mallone R Funaro A Zubiaur M Baj G Ausiello CM Tacchetti C. Signaling through CD38 induces NK cell activation. Int Immunol. (2001) 13:397–409. 10.1093/intimm/13.4.397 11282979

[46] Sconocchia G Titus JA Mazzoni A Visintin A Pericle F Hicks SW. CD38 triggers cytotoxic responses in activated human natural killer cells. Blood. (1999) 94:3864–71. 10572102

[47] Muñoz P Mittelbrunn M de la Fuente H Pérez-Martínez M García-Pérez A Ariza-Veguillas A. Antigen-induced clustering of surface CD38 and recruitment of intracellular CD38 to the immunologic synapse. Blood. (2008) 111:3653–64. 10.1182/blood-2007-07-101600 18212246

[48] Koopman LA Kopcow HD Rybalov B Boyson JE Orange JS Schatz F. Human decidual natural killer cells are a unique NK cell subset with immunomodulatory potential. J Exp Med. (2003) 198:1201–12. 10.1084/jem.20030305 14568979 PMC2194228

[49] Carlino C Stabile H Morrone S Bulla R Soriani A Agostinis C. Recruitment of circulating NK cells through decidual tissues: a possible mechanism controlling NK cell accumulation in the uterus during early pregnancy. Blood. (2008) 111:3108–15. 10.1182/blood-2007-08-105965 18187664

[50] Keskin DB Allan DSJ Rybalov B Andzelm MM Stern JNH Kopcow HD. TGFβ promotes conversion of CD16+ peripheral blood NK cells into CD16– NK cells with similarities to decidual NK cells. Proc Natl Acad Sci USA. (2007) 104:3378–83. 10.1073/pnas.0611098104 17360654 PMC1805591

[51] Goodridge JP Lathbury LJ John E Charles AK Christiansen FT Witt CS The genotype of the NK cell receptor, KIR2DL4, influences INF secretion by decidual natural killer cells. Mol Hum Reproduct. (2009) 15:489–97. 10.1093/molehr/gap039 19509110

[52] Li C Houser BL Nicotra ML Strominger JL. HLA-G homodimer-induced cytokine secretion through HLA-G receptors on human decidual macrophages and natural killer cells. Proc Natl Acad Sci USA. (2009) 106:5767–72. 10.1073/pnas.0901173106 19304799 PMC2667005

[53] Rajagopalan S Bryceson YT Kuppusamy SP Geraghty DE van der Meer A Joosten I. Activation of NK cells by an endocytosed receptor for soluble HLA-G. PLoS Biol. (2005) 4:e9. 10.1371/journal.pbio.0040009 16366734 PMC1318474

[54] Gamliel M Goldman-Wohl D Isaacson B Gur C Stein N Yamin R. Trained memory of human uterine NK cells enhances their function in subsequent pregnancies. Immunity. (2018) 48:951–62.e5. 10.1016/j.immuni.2018.03.030 29768178

[55] Glasner A Isaacson B Viukov S Neuman T Friedman N Mandelboim M. Increased NK cell immunity in a transgenic mouse model of NKp46 overexpression. Sci Rep. (2017) 7:13090. 10.1038/s41598-017-12998-w 29026144 PMC5638832

[56] Shi L Li K Guo Y Banerjee A Wang Q Lorenz UM. Modulation of NKG2D, NKp46, and Ly49C/I facilitates natural killer cell-mediated control of lung cancer. Proc Natl Acad Sci USA. (2018) 115:11808–13. 10.1073/pnas.1804931115 30381460 PMC6243255

[57] Han B Mao F-Y Zhao Y-L Lv Y-P Teng Y-S Duan M. Altered NKp30, NKp46, NKG2D, and DNAM-1 expression on circulating NK cells is associated with tumor progression in human gastric cancer. J Immunol Res. (2018) 2018:6248590. 10.1155/2018/6248590 30255106 PMC6140275

[58] Ichikawa A Kuba K Morita M Chida S Tezuka H Hara H. CXCL10-CXCR3 enhances the development of neutrophil-mediated fulminant lung injury of viral and nonviral origin. Am J Respirat Critic Care Med. (2013) 187:65–77. 10.1164/rccm.201203-0508OC 23144331 PMC3927876

[59] Soudja SM Ruiz AL Marie JC Lauvau G. Inflammatory monocytes activate memory CD8 T and innate NK lymphocytes independent of cognate antigen during microbial pathogen invasion. Immunity. (2012) 37:549–62. 10.1016/j.immuni.2012.05.029 22940097 PMC3456987

[60] Arruvito L Giulianelli S Flores AC Paladino N Barboza M Lanari C. NK cells expressing a progesterone receptor are susceptible to progesterone-induced apoptosis. J Immunol. (2008) 180:5746–53. 10.4049/jimmunol.180.8.5746 18390760

[61] Nilsson N Carlsten H. Estrogen induces suppression of natural killer cell cytotoxicity and augmentation of polyclonal B cell activation. Cell Immunol. (1994) 158:131–9. 10.1006/cimm.1994.1262 8087860

[62] Le Gars M Seiler C Kay AW Bayless NL Starosvetsky E Moore L CD38 contributes to human natural killer cell responses through a role in immune synapse formation. bioRxiv [Preprint]. 10.1101/349084

