# Supplementary material for: Pregnancy-Induced Alterations in NK Cell Phenotype and Function
Source: Front Immunol. 2019 Oct 23;10:2469. doi: 10.3389/fimmu.2019.02469 (PMC6820503; doi:10.3389/fimmu.2019.02469)
Supplement: Supplementary file 1 [file Table_1.DOCX]

**Supplementary tables.**

**Table S1** Demographics for discovery cohort

| **Characteristic** | **Pregnant, n = 21** | **Control, n=21** |
| --- | --- | --- |
| **^#^Age, years (median)** | *30.0 (21 to 42)* | *27.2 (19 to 43)* |
| **White, n (%)** | *4 (19)* | 11 (52) |
| **Asian, n (%)** | *4 (19)* | *5 (24)* |
| **Hispanic, n (%)** | *9 (43)* | *3 (14)* |
| **Race, other, n (%)** | *4 (19)* | *1 (5)* |
| **Trimester 2, n (%)** | *10 (48)* |  |
| **Trimester 3, n (%)** | *11 (52)* |  |

^#^ No significant differences were found between the age of control and pregnant women.

**Table S2** Demographics for validation cohort

| **Characteristic** | **Pregnant, n = 21** | **Control, n=32** |
| --- | --- | --- |
| **^#^Age, years (median)** | *29.2 (19 to 40)* | *36.8 (19 to 44)* |
| **White, n (%)** | *4 (19)* | 20 (62) |
| **Asian, n (%)** | *4 (19)* | *6 (18)* |
| **Hispanic, n (%)** | *9 (43)* | *5 (15)* |
| **Race, other, n (%)** | *4 (19)* | *1(3)* |
| **Trimester 2, n (%)** | *10 (48)* |  |
| **Trimester 3, n (%)** | *11 (52)* |  |

^#^ No significant differences were found between the age of control and pregnant women.

**Table S3.** Antibody panel for mass cytometry in discovery cohort.

| Isotope | Antigen | Clone |
| --- | --- | --- |
| 112Cd | HLA-DR | Tu36 |
| 115In | CD20 | 2H7 |
| 141Pr | CD38 | HIT2 |
| 142Nd | CD3 | UCHT1 |
| 145Nd | CD57 | HCD57 |
| 146Nd | CD27 | O323 |
| 149Sm | CD19 | HIB19 |
| 150Nd | CD14 | M5E2 |
| 154Sm | PD1 | EH12.2H7 |
| 155Gd | NKp46 | 9E2/NKp46 |
| 157Gd | NKG2C | 134591 |
| 159Tb | CD16 | 3G8 |
| 160Gd | CXCR3 | G025H7 |
| 161Dy | NKp30 | P30-15 |
| 163Dy | NKp44 | P44-8 |
| 171Yb | NKG2A | Z199 |
| 172Yb | NKG2D | 1D11 |
| 173Yb | CXCR5 | 51505 |
| 174Yb | CD56 | NCAM16.2 |
| 176Yb | CD25 | M-A251 |

**Table S4.** Antibody panel for mass cytometry in validation cohort.

| Isotope | Antigen | Clone |
| --- | --- | --- |
| 115Ln | CD20 | 2H7 |
| 141Pr | CD38 | HIT2 |
| 142Nd | CD11b | ICRF44 |
| 146Nd | CD27 | O323 |
| 149Sm | CD16 | 3G8 |
| 150Nd | CD14 | M5E2 |
| 151Eu | CD19 | HIB19 |
| 152Sm | CD15 | W6D3 |
| 154Gd | LILRB1 | GHI/75 |
| 155Gd | NKp46 | 9E2/NKp46 |
| 156Gd | CD3 | UCHT1 |
| 157Gd | NKG2C | 134591 |
| 158Gd | CD244 | Feb-69 |
| 159Tb | CD33 | WM53 |
| 161Dy | NKp30 | P30-15 |
| 164Dy | NKp44 | P44-8 |
| 166Er | KIR2DL1 | 143211 |
| 167Er | CD94 | DX22 |
| 168Er | CXCR6 | 56811 |
| 170Yb | KIR2DL3 | 180701 |
| 171Yb | NKG2A | Z199 |
| 172Yb | NKG2D | 1D11 |
| 173Yb | KIR2DL4 | mAB 33 |
| 174Yb | CD56 | NCAM16.2 |
| 176Yb | CD25 | M-A251 |
| 191lr | DNA1 |  |
| 193lr | DNA2 |  |
| 195Pt | Cisplatin |  |
