# Supplementary material for: Pregnancy-Induced Alterations in NK Cell Phenotype and Function
Source: Front Immunol. 2019 Oct 23;10:2469. doi: 10.3389/fimmu.2019.02469 (PMC6820503; doi:10.3389/fimmu.2019.02469)
Supplement: Supplementary file 2 [file Data_Sheet_1.PDF]

Figure S1

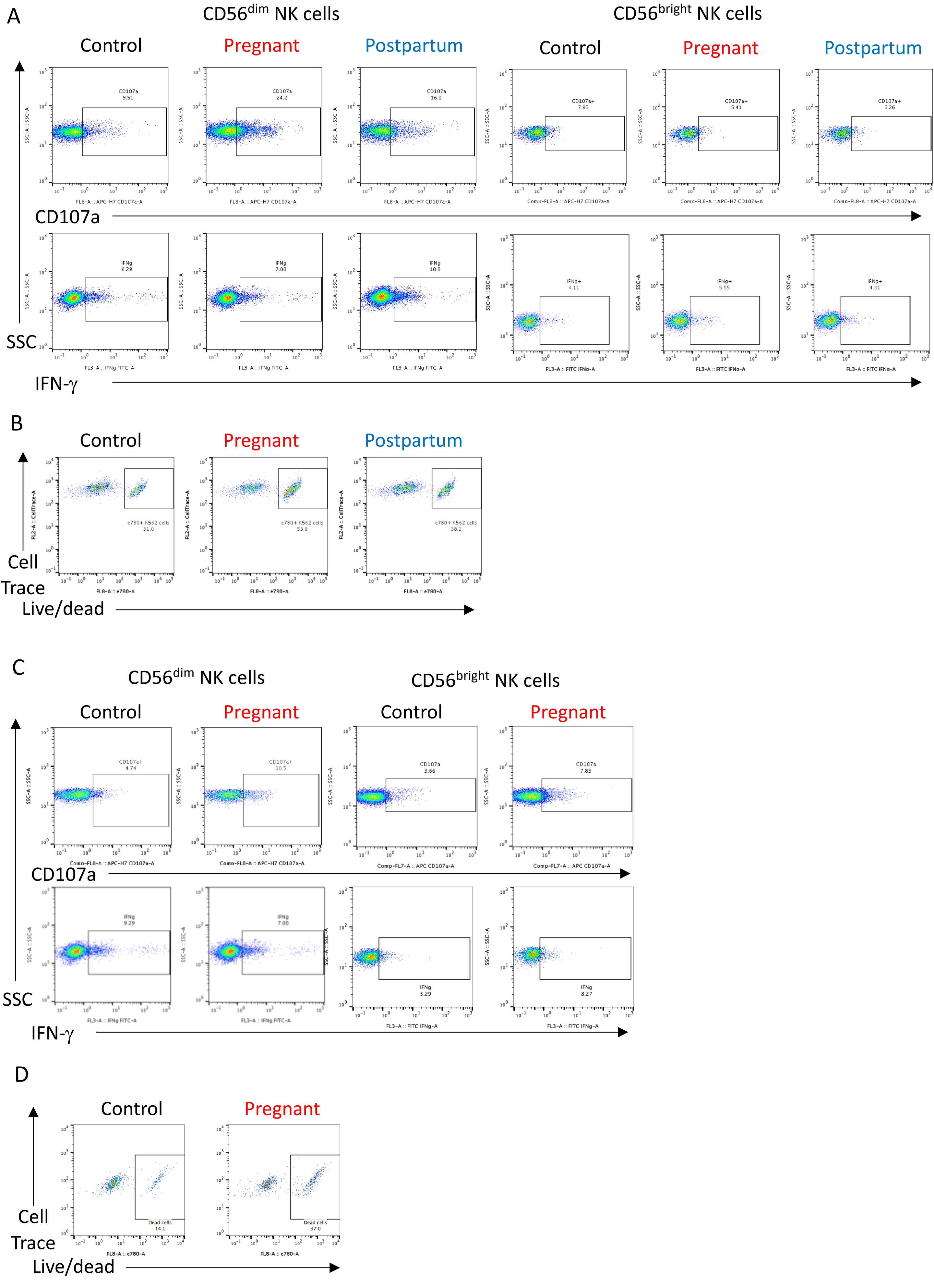
A
CD56dim NK cells
CD56bright NK cells
Control
Pregnant
Postpartum
CD107a
9.51
24.2
16.0
CD107a+
7.93
5.41
5.26
IFNg
9.29
7.00
10.8
IFNg+
4.11
5.55
4.31
SSC
IFN-γ
B
Cell Trace
Live/dead
e780+ K562 cells
31.0
53.8
38.2
C
4.74
10.5
3.66
7.83
5.29
8.27
D
Dead cells
14.1
37.0

Figure S1. CD56^{dim} and CD56^{bright} NK cell immune response to influenza infected and tumor cells during pregnancy.

(A) Representative plots showing the the frequency of (A) CD107a- and IFN-γ-expressing CD56^{dim} and CD56^{bright} NK cells from controls, pregnant and postpartum women in response to influenza-infected monocytes. (B) The frequency of dead or dying influenza-infected monocytes per group is represented. (C) The frequency of CD107a and IFN-γ-positive by CD56^{dim} and CD56^{bright} NK cells in response to K562 cells is represented. (D) The frequency of dead or dying K562 tumor cells based on staining with viability dye in NK cell co-culture.

Figure S2

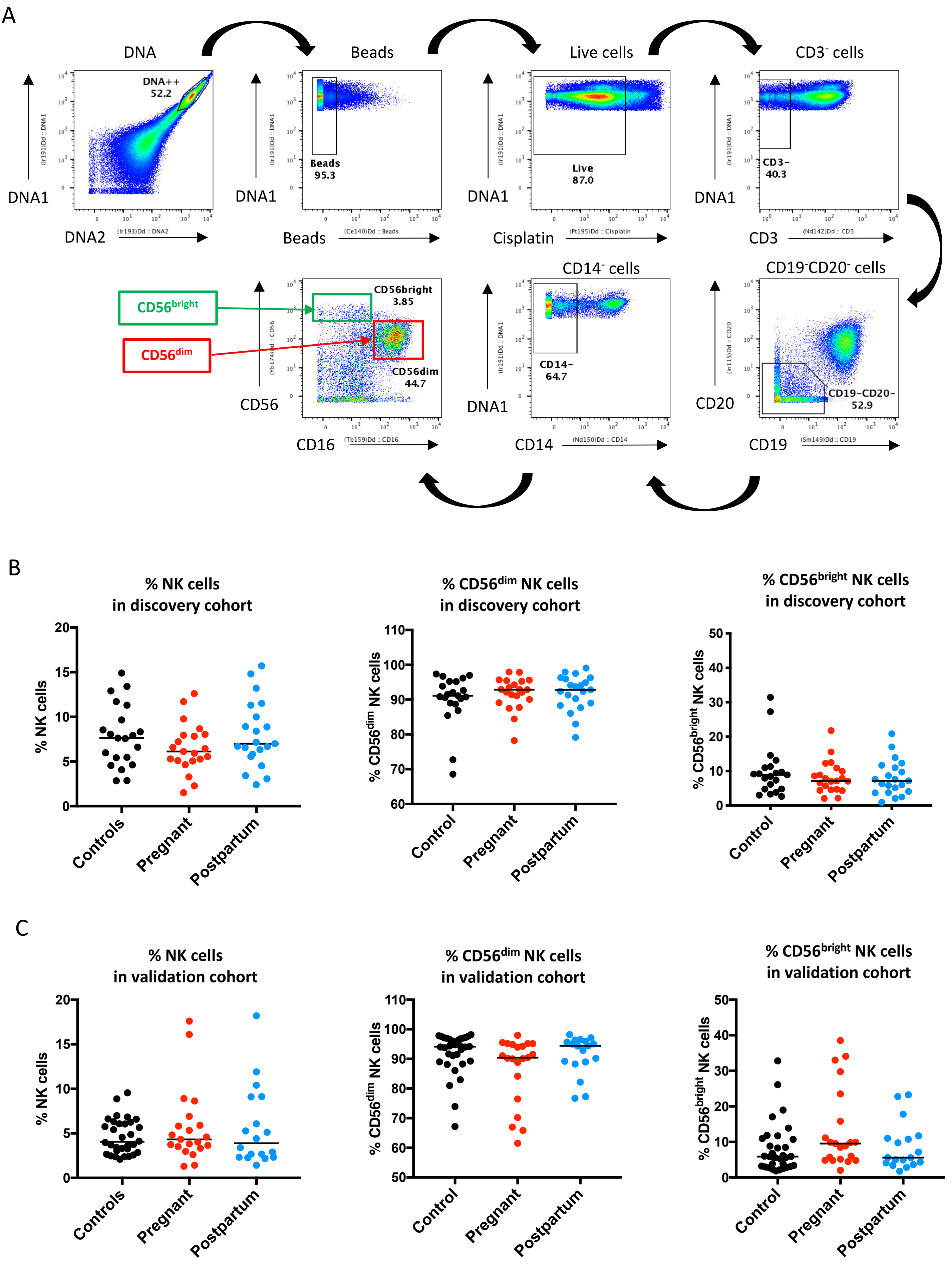
A
DNA
DNA++
52.2
DNA1
DNA2
Beads
Beads
95.3
Live cells
Live
87.0
Cisplatin
CD3- cells
CD3-
40.3
CD3
CD19-CD20- cells
CD19-CD20-
52.9
CD20
CD19
CD14- cells
CD14-
64.7
CD14
CD56bright
3.85
CD56dim
44.7
CD56bright
CD56dim
CD56
CD16
B
% NK cells
in discovery cohort
% CD56dim NK cells
in discovery cohort
% CD56bright NK cells
in discovery cohort
% NK cells
% CD56dim NK cells
% CD56bright NK cells
Controls
Control
Pregnant
Postpartum
C
% NK cells
in validation cohort
% CD56dim NK cells
in validation cohort
% CD56bright NK cells
in validation cohort

Figure S2. CD56^{dim} and CD56^{bright} NK cell gating strategy and proportion during pregnancy.

(A**)** Two-dimensional mass cytometry plots are shown for a representative patient sample. Gating was performed using FlowJo software (FlowJo, LLC). Negative lineage gating was performed to exclude CD3^{+} T cells, CD19^{+}CD20^{+} B cells and CD14^{+} monocytes, followed by a positive gating on CD56^{+}CD16$^{+/-}$, CD56^{dim} or CD56^{bright} NK cells. Percentage of total NK cells, and CD56^{dim} and CD56^{bright} NK cells within PBMCs of controls, pregnant women and postpartum in discovery (B) and validation (C) cohorts.

Figure S3

Gating strategy for each marker – CD56^{dim} NK cell population from discovery cohort

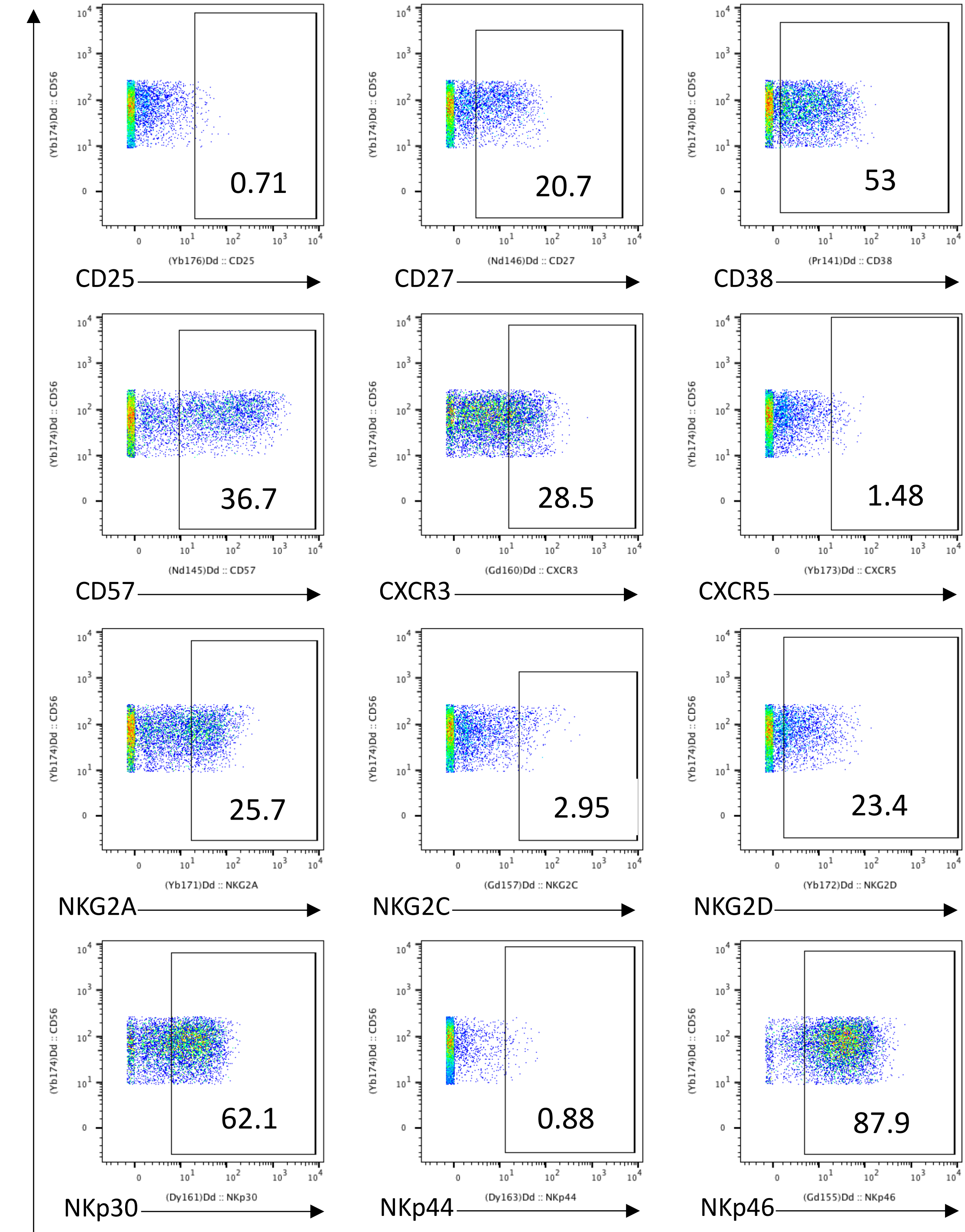

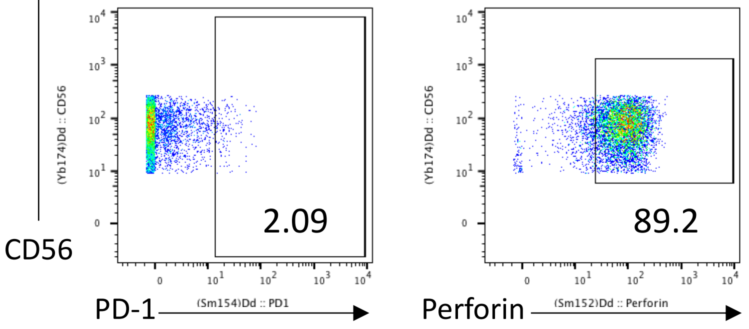

Figure S3. Gating strategy for each marker on CD56^{dim} NK cells from discovery cohort. Representative plots for each marker are shown. Numbers indicate the percent of positive cells per marker.

Figure S4

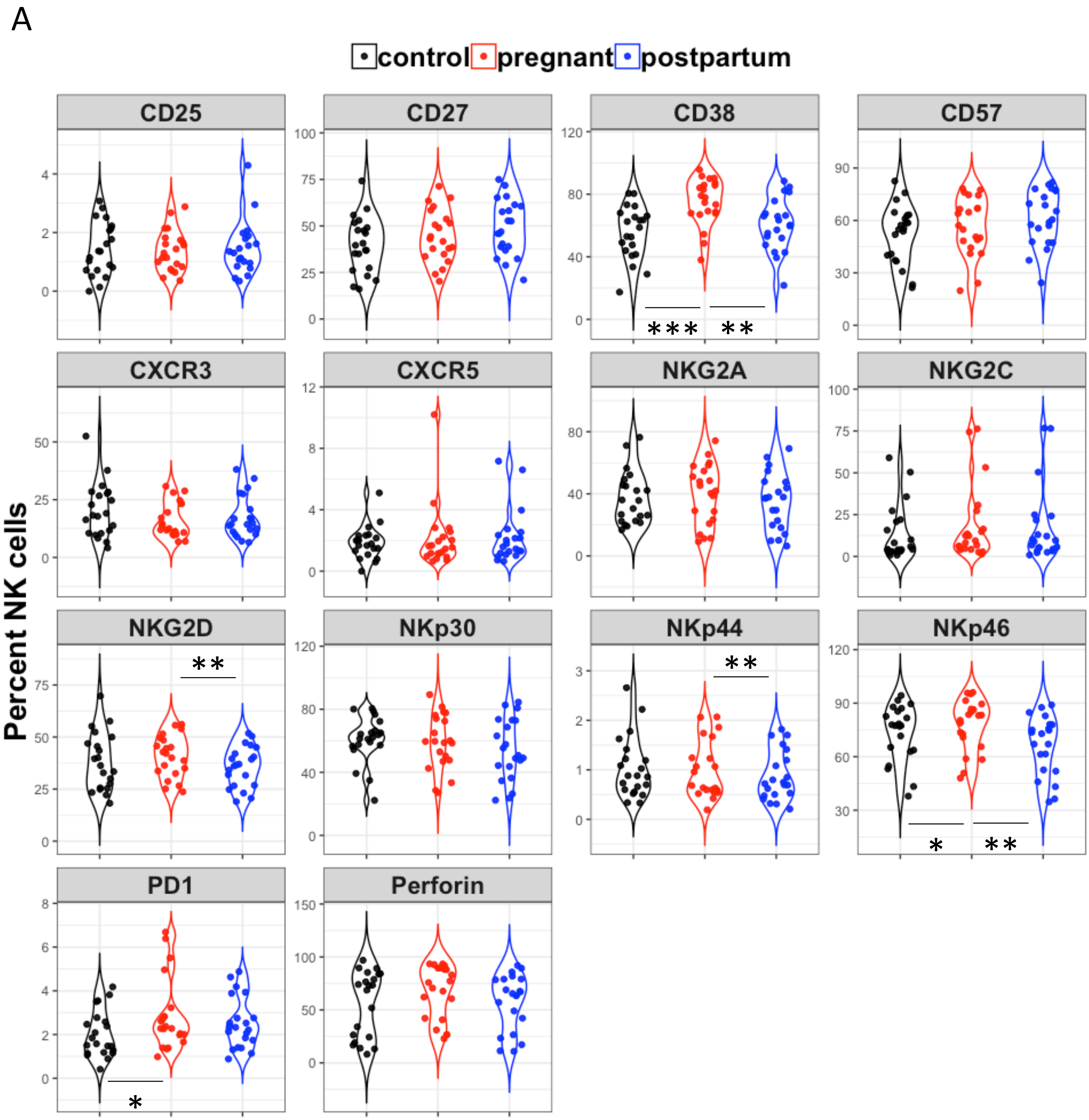
A
control
pregnant
postpartum
Percent NK cells
CD25
CD27
CD38
CD57
CXCR3
CXCR5
NKG2A
NKG2C
NKG2D
NKp30
NKp44
NKp46
PD1
Perforin
***
**
*

B

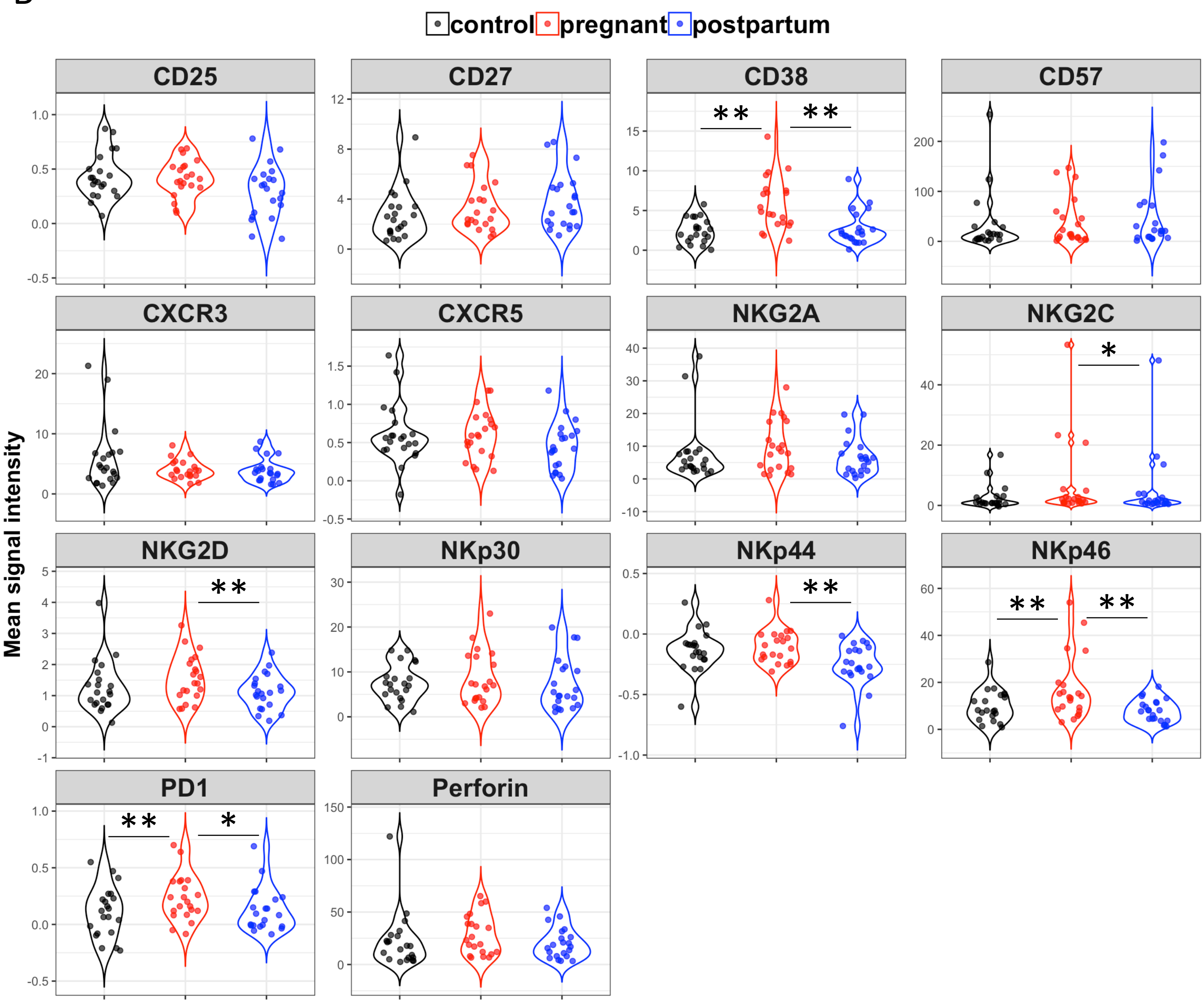

Figure S4. Percentage of expression for each marker on CD56^dim NK cells from discovery cohort using conventional gating.

NK cells from controls and pregnant women, as well as postpartum on discovery cohort were isolated and stained using a 14-parameter antibody panel, respectively. Percentage (A) and mean signal intensity (B) of each marker on CD56^dim NK cells was determined by conventional gating for discovery cohort. *P<0.05, **P<0.01 and ***P<0.001 (Mann–Whitney *U* Tests to compare controls vs. pregnant; Wilcoxon matched-paired test to compare pregnant vs. postpartum).

Figure S5

Gating strategy for each marker – CD56^{bright} NK cell population from discovery cohort

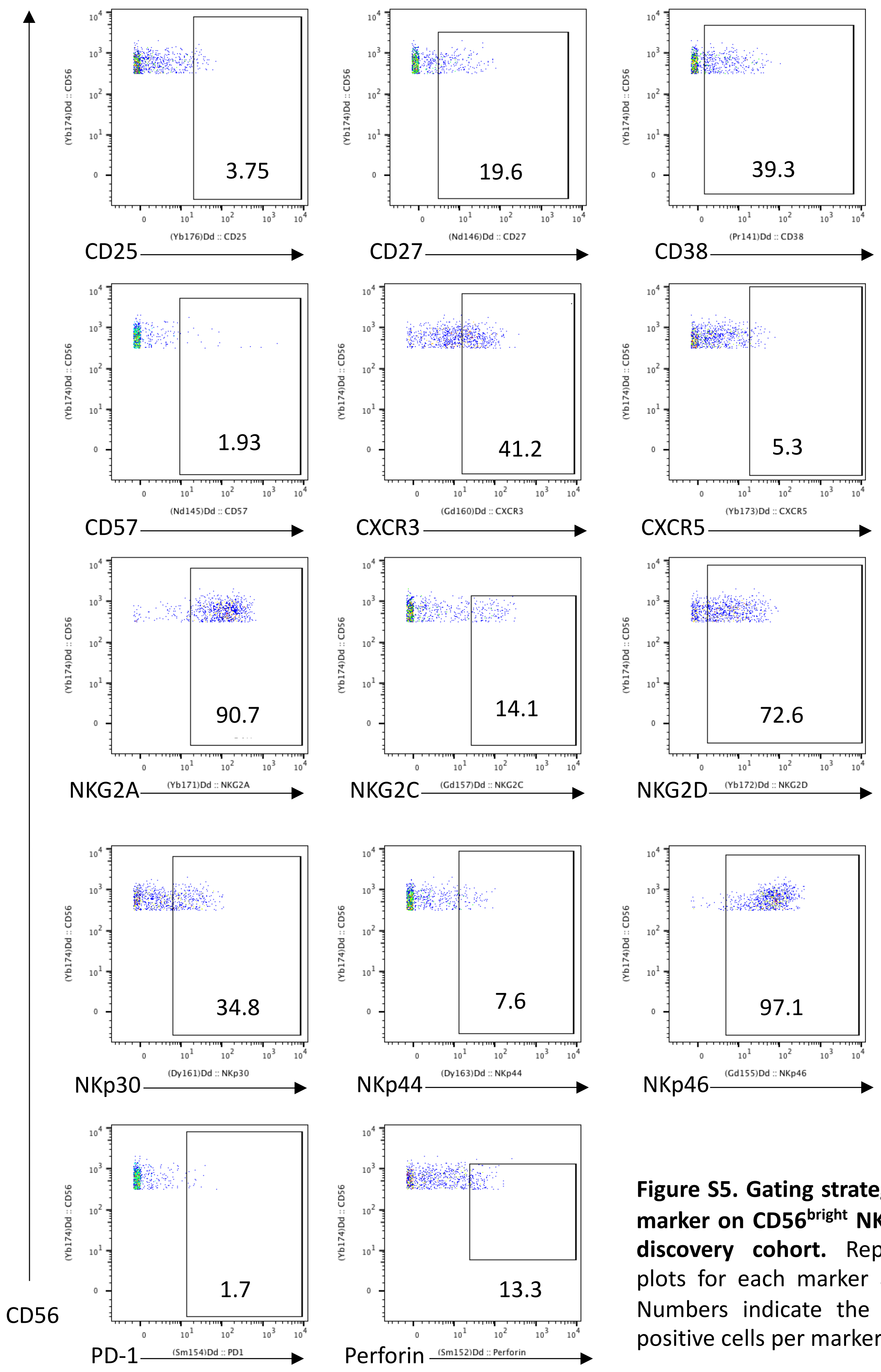

Figure S5. Gating strategy for each marker on CD56^{bright} NK cells from discovery cohort. Representative plots for each marker are shown. Numbers indicate the percent of positive cells per marker.

Figure S6

A

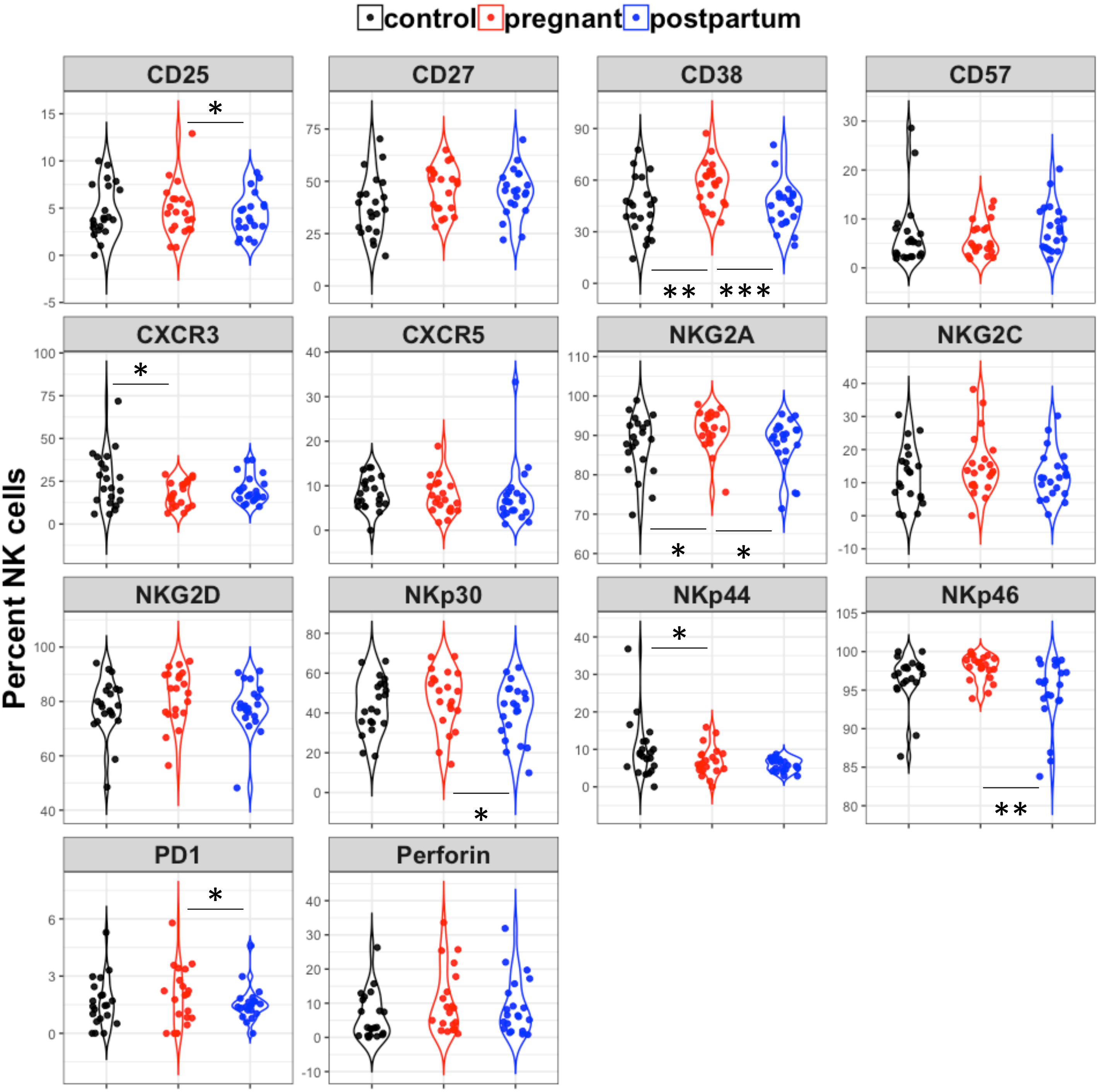

control
pregnant
postpartum
Percent NK cells
CD25
CD27
CD38
CD57
CXCR3
CXCR5
NKG2A
NKG2C
NKG2D
NKp30
NKp44
NKp46
PD1
Perforin

B

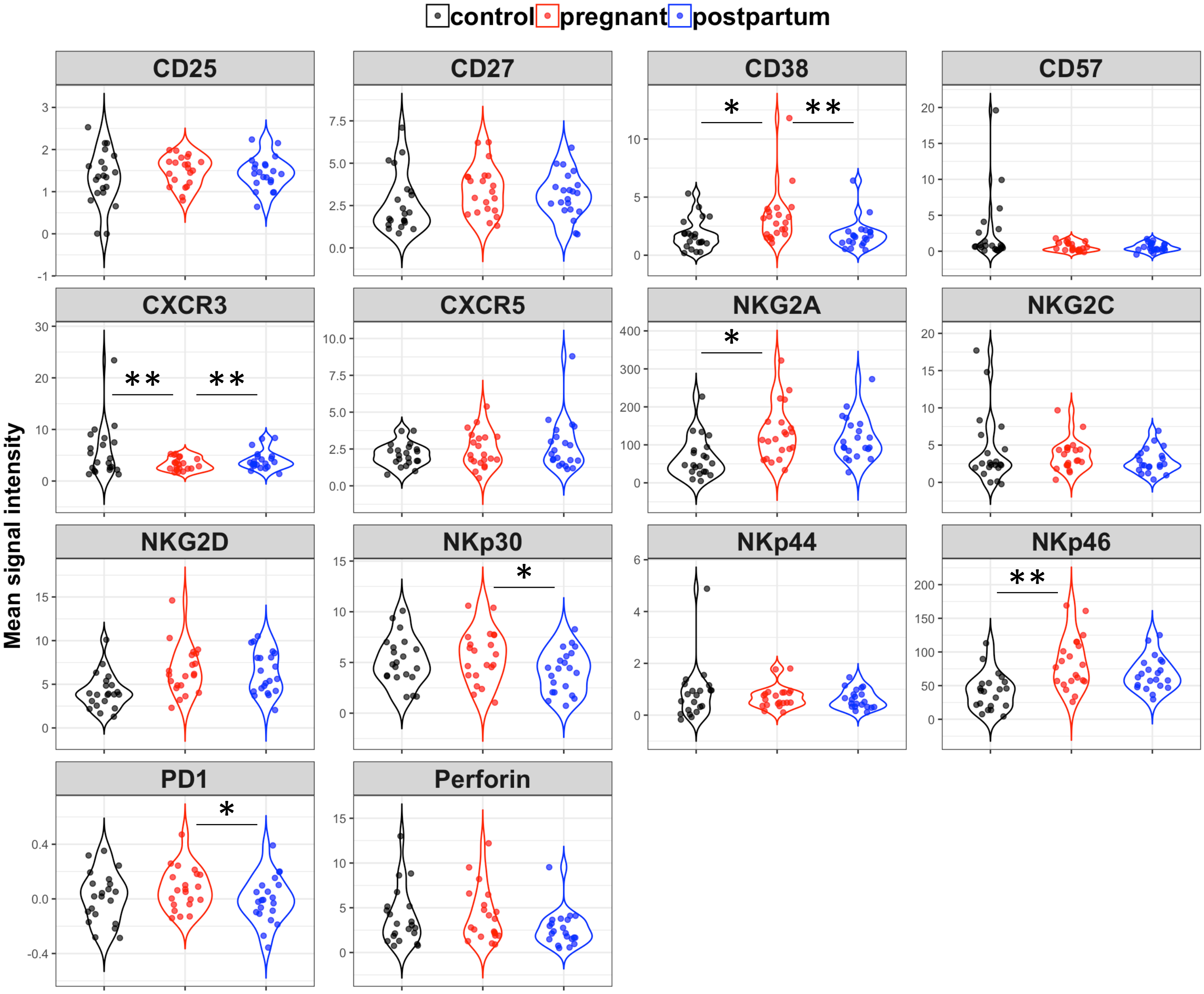

Figure S6. Percentage of expression for each marker on CD56^bright NK cells from discovery cohort using conventional gating.

NK cells from controls and pregnant women, as well as postpartum on discovery cohort were isolated and stained using a 14-parameter antibody panel, respectively. Percentage (A) and mean signal intensity (B) of each marker on CD56^bright NK cells was determined by conventional gating for discovery cohort. *P<0.05, **P<0.01 and ***P<0.001 (Mann–Whitney *U* Tests to compare controls vs. pregnant; Wilcoxon matched-paired test to compare pregnant vs. postpartum).

Figure S7

Gating strategy for each marker – CD56^{dim} NK cell population from validation cohort

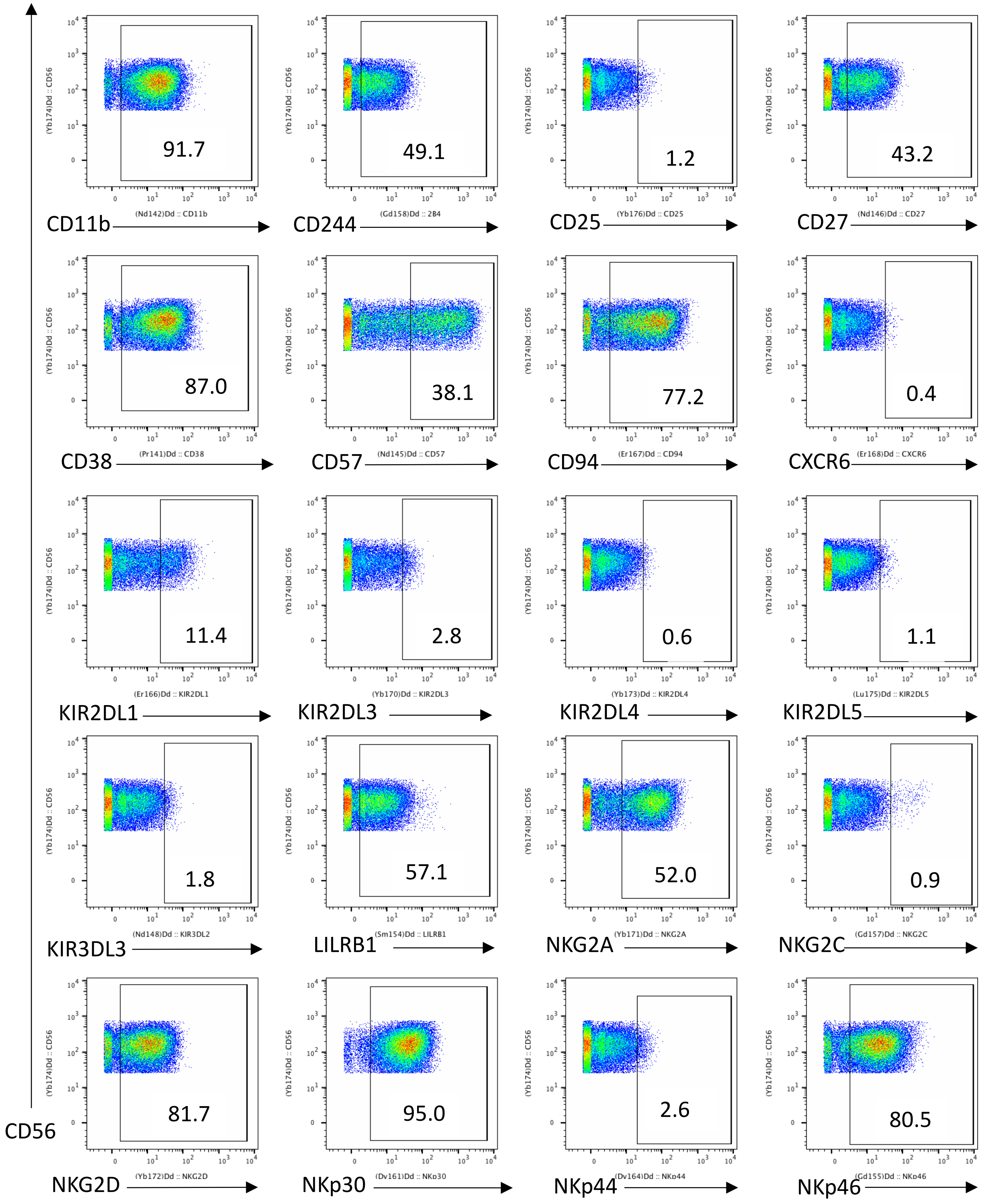

Figure S7. Gating strategy for each marker on CD56^{dim} NK cells from validation cohort. Representative plots for each marker are shown. Numbers indicate the percent of positive cells per marker.

Figure S8

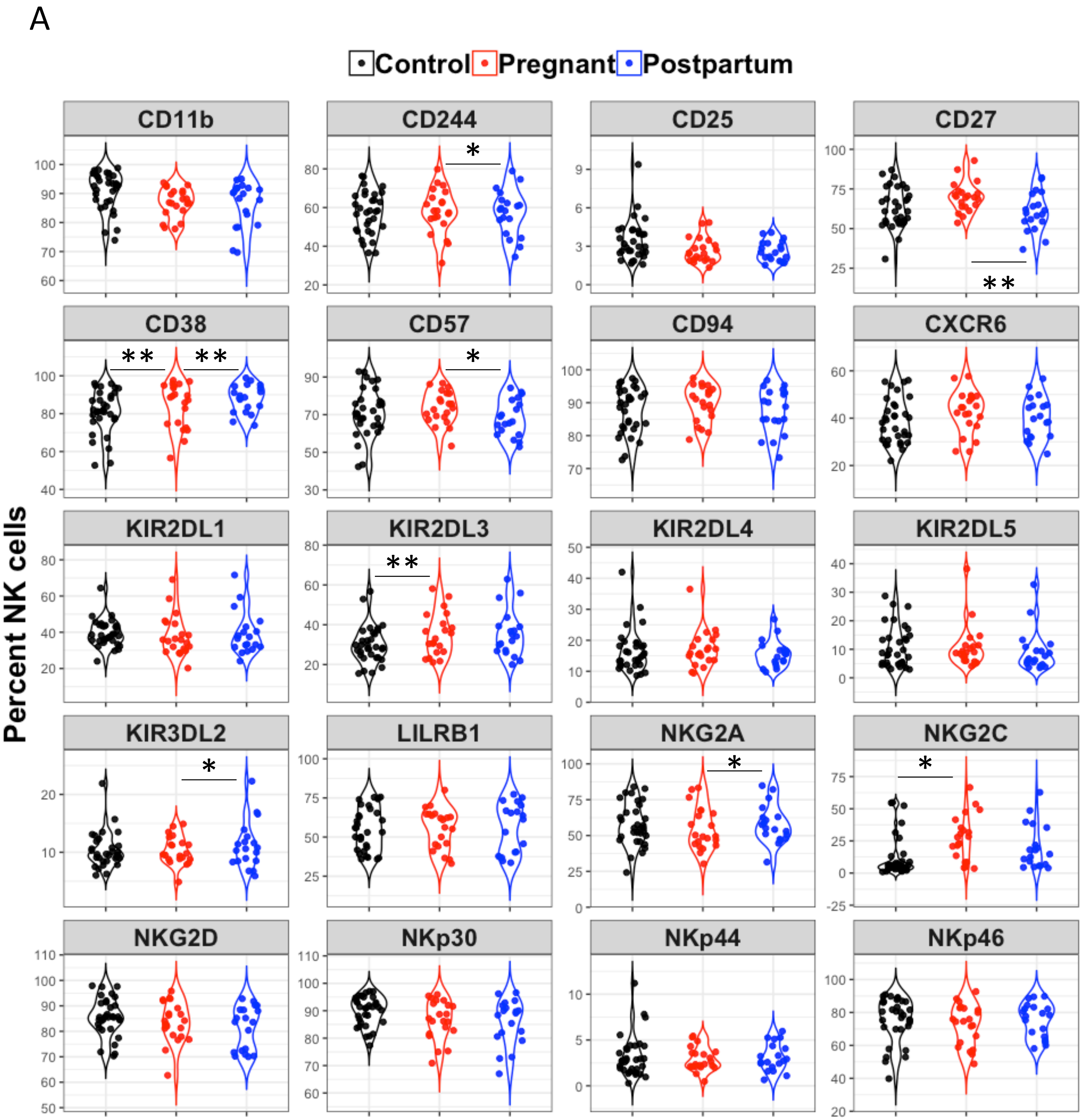
A
Control
Pregnant
Postpartum
Percent NK cells
CD11b
CD244
CD25
CD27
CD38
CD57
CD94
CXCR6
KIR2DL1
KIR2DL3
KIR2DL4
KIR2DL5
KIR3DL2
LILRB1
NKG2A
NKG2C
NKG2D
NKp30
NKp44
NKp46

B

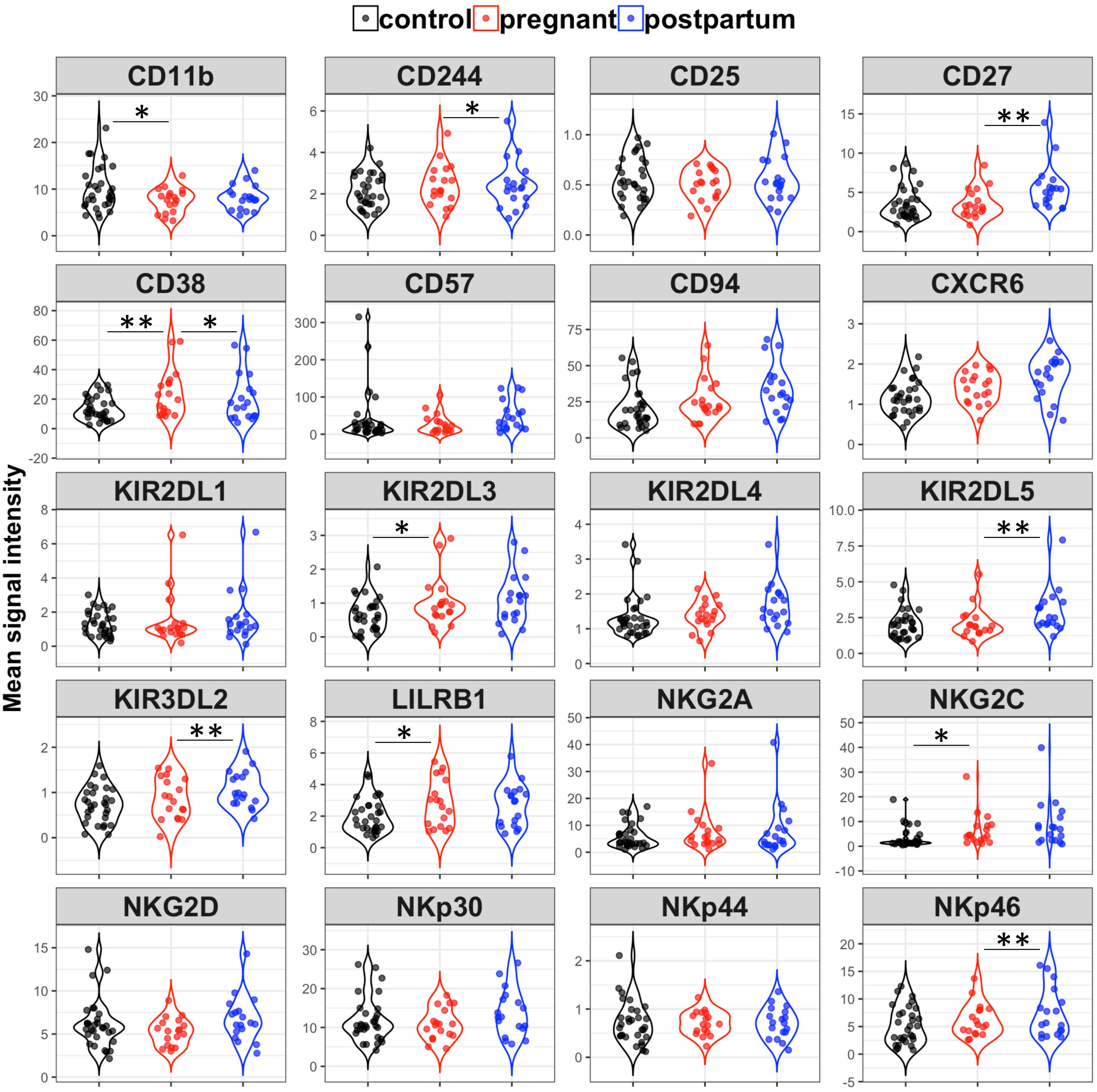

Figure S8. Percentage of expression for each marker on CD56^{dim} NK cells from discovery cohort using conventional gating.

NK cells from controls and pregnant women, as well as postpartum on validation cohort were isolated and stained using a 20-parameter antibody panel, respectively. Percentage (A) and mean signal intensity (B) of each marker on CD56^{dim} NK cells was determined by conventional gating for validation cohort. *P<0.05, **P<0.01 and ***P<0.001 (Mann–Whitney *U* Tests to compare controls vs. pregnant; Wilcoxon matched-paired test to compare pregnant vs. postpartum).

Figure S9

Gating strategy for each marker – CD56^{bright} NK cell population from validation cohort

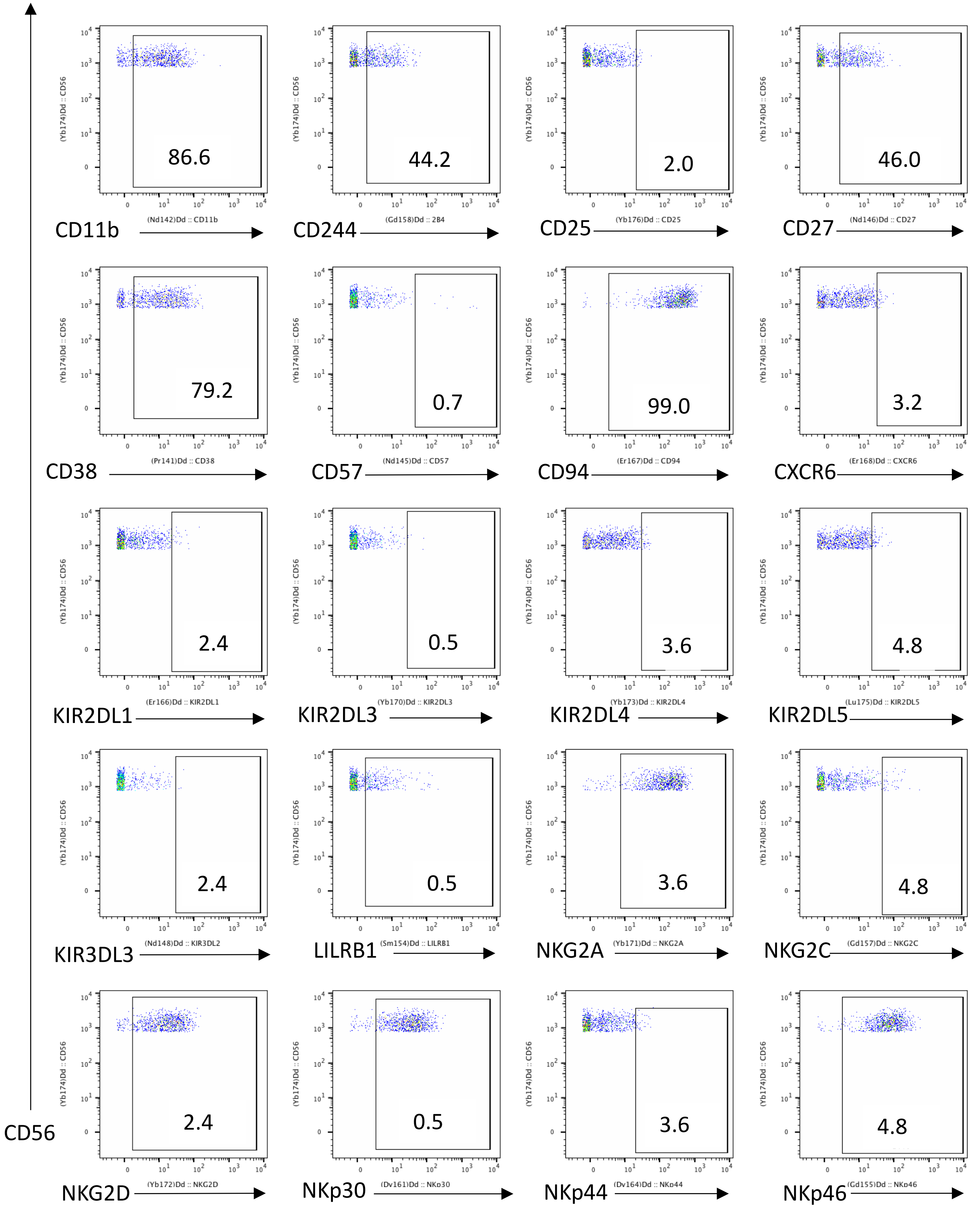

Figure S9. Gating strategy for each marker on CD56^{bright} NK cells from validation cohort. Representative plots for each marker are shown. Numbers indicate the percent of positive cells per marker.

Figure S10

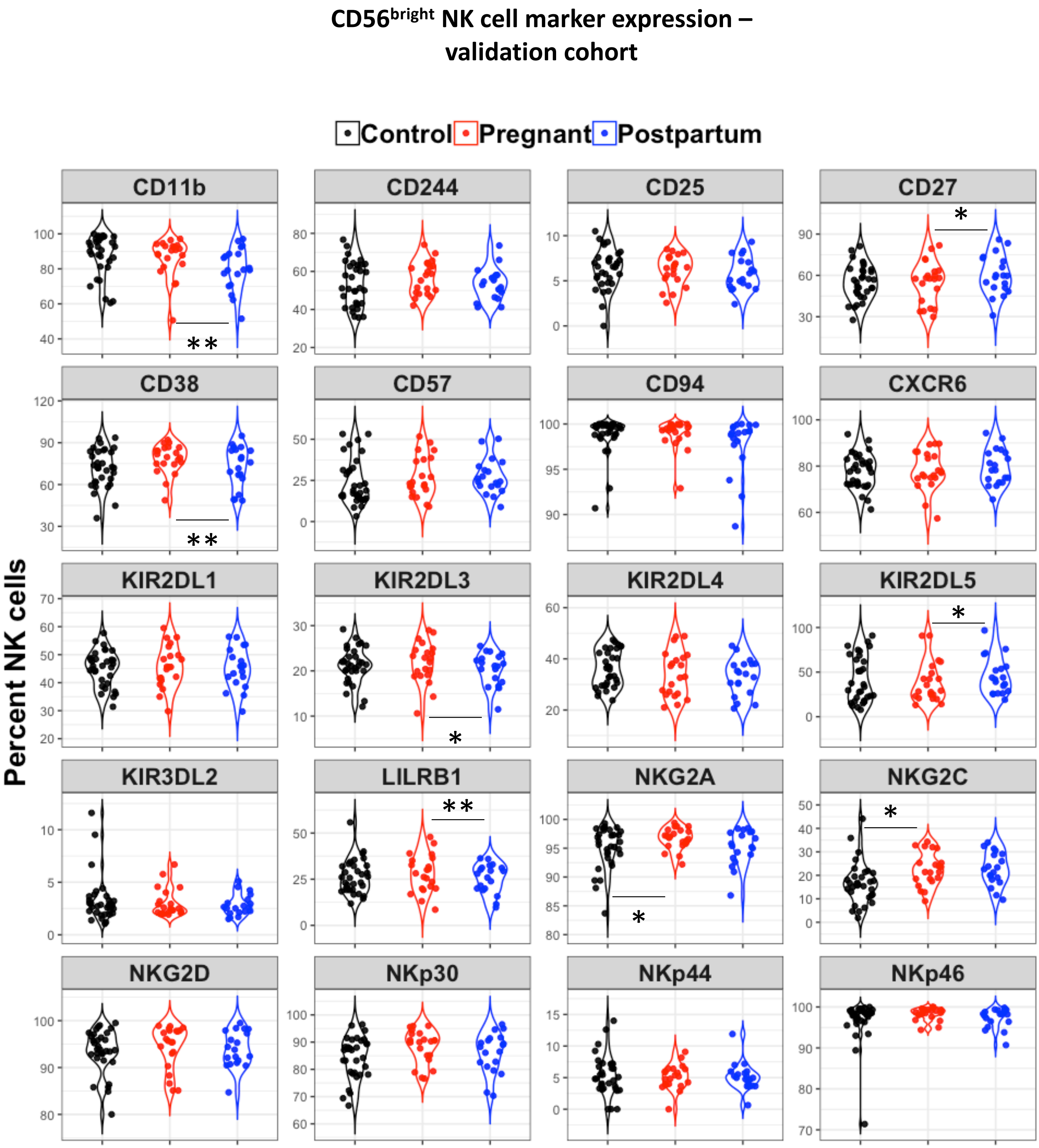
CD56bright NK cell marker expression – validation cohort
Control
Pregnant
Postpartum
Percent NK cells
CD11b
CD244
CD25
CD27
CD38
CD57
CD94
CXCR6
KIR2DL1
KIR2DL3
KIR2DL4
KIR2DL5
KIR3DL2
LILRB1
NKG2A
NKG2C
NKG2D
NKp30
NKp44
NKp46

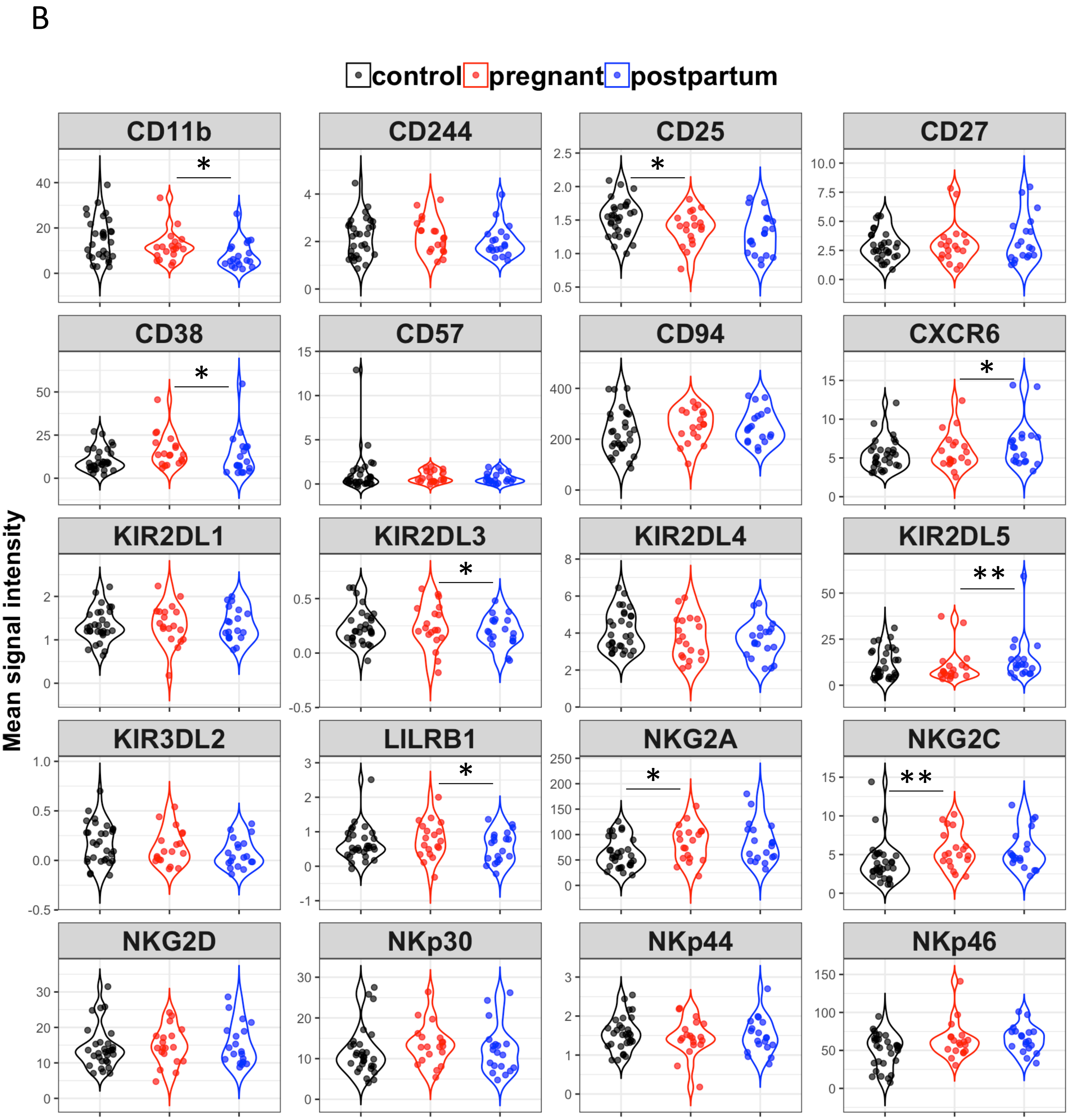

Figure S10. Percentage of expression for each marker on CD56^{bright} NK cells from validation cohort using conventional gating.

NK cells from controls and pregnant women, as well as postpartum on validation cohort were isolated and stained using a 20-parameter antibody panel, respectively. Percentage (A) and mean signal intensity (B) of each marker on CD56^{bright} NK cells was determined by conventional gating for validation cohort. *P<0.05 and **P<0.01 (Mann–Whitney *U* Tests to compare controls vs. pregnant; Wilcoxon matched-paired test to compare pregnant vs. postpartum).

Figure S11

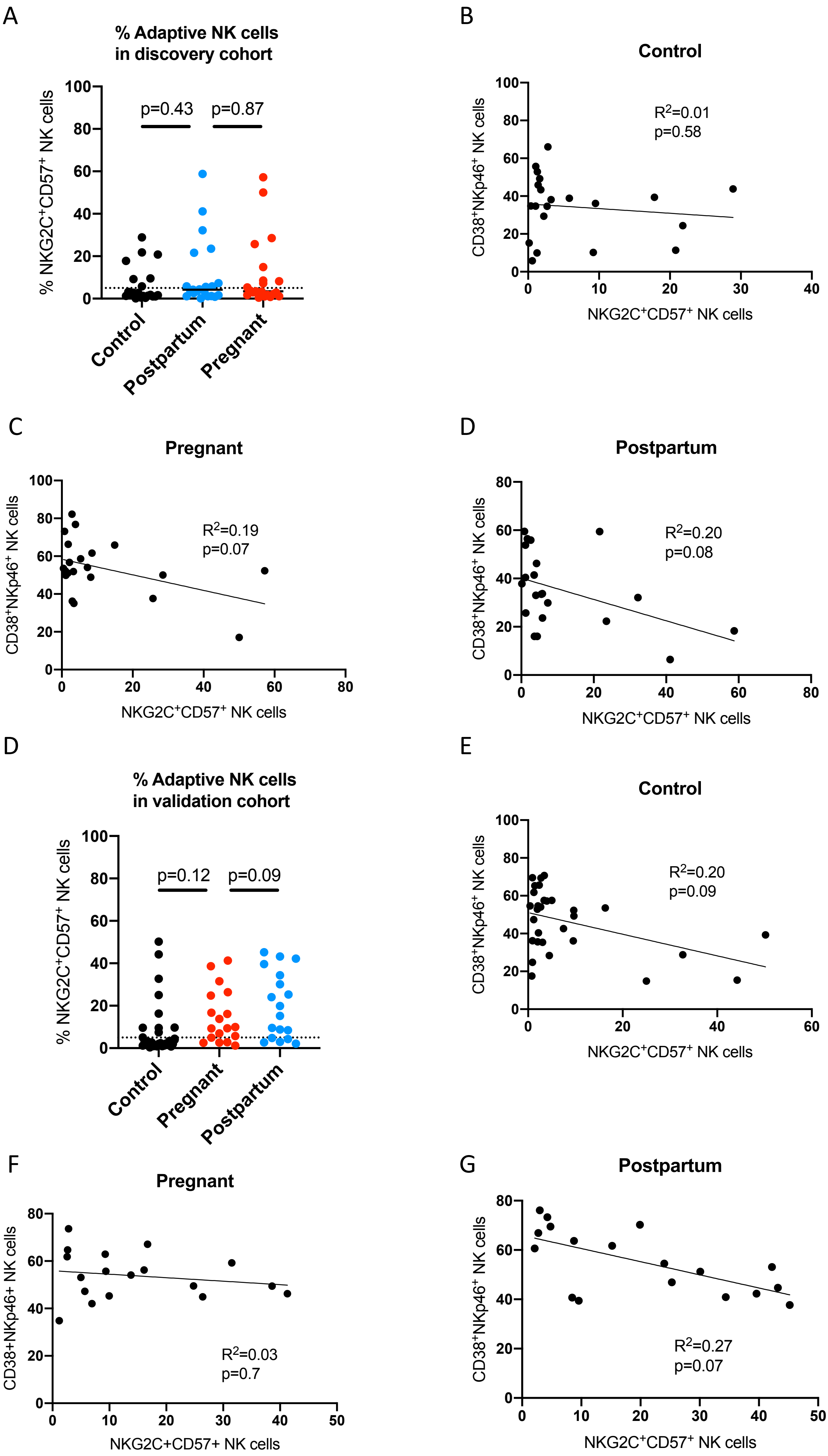
A
% Adaptive NK cells
in discovery cohort
% NKG2C+CD57+ NK cells
p=0.43
p=0.87
Control
Postpartum
Pregnant
B
Control
CD38+NKp46+ NK cells
R2=0.01
p=0.58
NKG2C+CD57+ NK cells
C
Pregnant
CD38+NKp46+ NK cells
R2=0.19
p=0.07
NKG2C+CD57+ NK cells
D
Postpartum
CD38+NKp46+ NK cells
R2=0.20
p=0.08
NKG2C+CD57+ NK cells
D
% Adaptive NK cells
in validation cohort
% NKG2C+CD57+ NK cells
p=0.12
p=0.09
Control
Pregnant
Postpartum
E
Control
CD38+NKp46+ NK cells
R2=0.20
p=0.09
NKG2C+CD57+ NK cells
F
Pregnant
CD38+NKp46+ NK cells
R2=0.03
p=0.7
NKG2C+CD57+ NK cells
G
Postpartum
CD38+NKp46+ NK cells
R2=0.27
p=0.07
NKG2C+CD57+ NK cells

Figure S11. Percent of adaptive NK cells and correlation with $CD38^{+}NKp46^{+}$ NK cells between controls, pregnant and postpartum in the discovery and validation cohorts.

(A) Percent of adaptive $NKG2C^{+}CD57^{+}$ NK cells in control, pregnant and postpartum women from discovery cohort. Correlation between the percent of NKG2C+CD57+ NK cells and $CD38^{+}NKp46^{+}$ NK cells for controls ((B), pregnant (C) and postpartum (D) from discovery cohort. (E) Percent of adaptive $NKG2C^{+}CD57^{+}$ NK cells in control, pregnant and postpartum women from validation cohort. Correlation between the percent of $NKG2C^{+}CD57^{+}$ NK cells and $CD38^{+}NKp46^{+}$ NK cells for controls (F), pregnant (G) and postpartum (H) from validation cohort.
